# Ligand-Free MgCO_3_ Nanoclusters Catalyze Nucleophilic Alcohol Addition Reactions

**DOI:** 10.1021/acsami.5c21329

**Published:** 2026-02-10

**Authors:** Lluís Martínez-Belenguer, Kateřina Zítová, Jose Pedro Cerón-Carrasco, Belén Lerma-Berlanga, Antonio Leyva-Pérez

**Affiliations:** † Instituto de Tecnología Química, Universitat Politècnica de València-Agencia Estatal Consejo Superior de Investigaciones Científicas, Avda. de los Naranjos s/n, 46022 Valencia, Spain; ‡ Department of Organic Technology, University of Chemistry and Technology Prague, Technická 5, 16628 Prague 6, Czech Republic; § Centro Universitario de la Defensa, Academia General del Aire, 16769 Universidad Politécnica de Cartagena, C/Coronel López Peña S/N, Santiago de La Ribera, 30720 Murcia, Spain

**Keywords:** magnesium carbonate, nanoparticles, catalysis, alcohols, esters

## Abstract

Subnano and nanometric metal clusters are ultrasmall aggregates in which most atoms are exposed on the surface, directly interacting with reactants and enabling highly efficient catalysis. However, metal carbonate clusters have been barely prepared and used in catalysis. Here, we report the synthesis of ultrasmall, ligand-free MgCO_3_ clusters formed via CO_2_ capture with MgCl_2_, with an average composition of [MgCO_3_]_5_·3H_2_O. These clusters exhibit catalytic activity in various nucleophilic alcohol addition reactions, showing a 5-fold enhancement compared to bulk MgCO_3_ and CaCO_3_–triethylamine clusters. These results pave the way for synthesis of ultrasmall alkaline metal carbonate clusters beyond Ca, which can be employed as efficient catalysts in organic synthesis.

## Graphical abstract

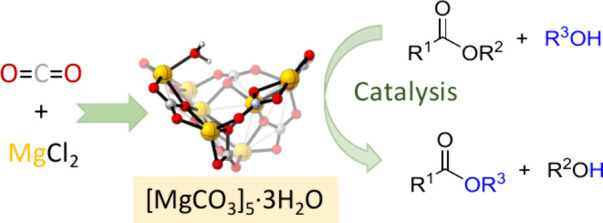

## Introduction

Alkaline carbonates constitute the main reservoir of carbon atoms on Earth, mainly in the form of MgCO_3_ and CaCO_3_,^1^ and one of the main strategies for climate change abatement consists of CO_2_ sequestration in the form of carbonates.^2^ Therefore, it is not surprising that MgCO_3_ and CaCO_3_ are among the cheapest and most nontoxic metal compounds worldwide. While this should spur the use of these salts as metal catalysts, particularly in organic synthesis, examples are just testimonial,^3^ and the bulk carbonates are usually relegated to be employed as a catalyst support, desiccants, or as a stoichiometric base.^4–6^

The low catalytic activity of alkaline carbonates in organic synthesis might be explained by the strong coordination of the carbonate anion to the divalent metal atom, the low surface area of the bulk material (with any proper structuration), and the easy hydration of the hard Lewis metal cation, which renders unsuitable solids for catalyzing organic reactions.^7^ However, these drawbacks could be all circumvented at once if the alkaline carbonate species are prepared in the form of subnanometric clusters.^8^ In this way, most (if not all) the metal carbonate atoms would place at the outer surface of the ultrasmall agglomerate, with a dynamic structure where uncoordinated positions can be found.^9,10^ Besides, the cluster becomes partially or totally soluble in organic solvents, available to readily interact with organic reagents.^11^

Scheme 1 (top) shows the recently reported synthesis of subnanometric CaCO_3_ clusters ([CaCO_3_]_*n*_).^12^ This synthesis consists of the capture of CO_2_ by hydrated CaCl_2_ salts in EtOH, in the presence of triethylamine (TEA), and proceeds at room temperature in just 40 min. TEA is used to quench the generated HCl and stabilize the clusters with N···H–O bonds, and, in this way, eight CaCO_3_ units in average (*n* = 8 for [CaCO_3_]_*n*_) conform stable entities with sizes below the nanometer.^12^ We have been able to reproduce this procedure and employ these [CaCO_3_]_8_ clusters as soluble supports/ligands for Pd single atoms and ultrasmall Pd clusters during the catalytic semi-hydrogenation of alkynes.^13^ The subnanometric [CaCO_3_]_*n*_ clusters have been further studied and applied in different fields by other groups.^14^

**Scheme 1:**
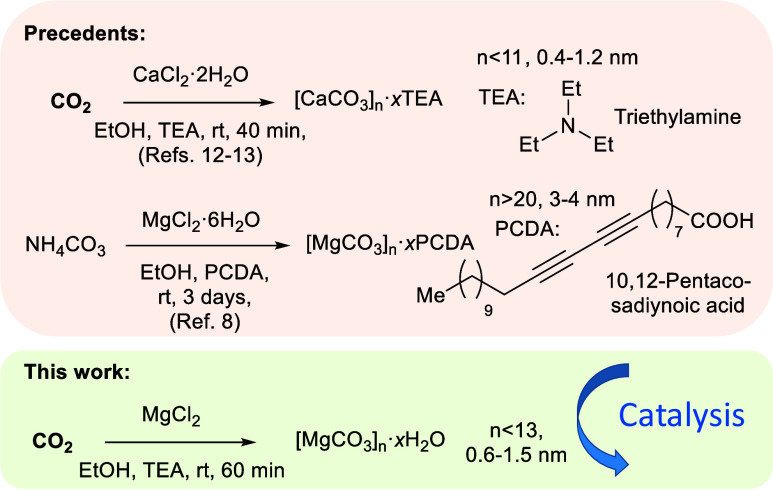
Some Representative Previous Works on the Synthesis of (Sub)­nanometric CaCO_3_ ([CaCO_3_]_*n*_) and MgCO_3_ ([MgCO_3_]_*n*_) Clusters (top) and Our Approach Here (bottom)

The [CaCO_3_]_8_ clusters are a good starting point to start the study of soluble metal carbonates as catalysts in organic synthesis. Since EtOH is the solvent of choice to prepare these ultrasmall clusters,^12^ we choose the nucleophilic addition of EtOH to different acceptors as reasonable transformations to be assessed with these [CaCO_3_]_8_ clusters as a potential catalyst.^15^ However, MgCO_3_ is a more ionized salt (Mg^2+^ is a harder cation than Ca^2+^ in virtue of its shorter radius)^16^ and, in principle, the corresponding MgCO_3_ clusters ([MgCO_3_]_*n*_) could be more catalytically active for ethanolysis reactions. Unfortunately, in contrast to subnanometric [CaCO_3_]_*n*_ clusters, synthetic procedures for [MgCO_3_]_*n*_ clusters are very scarce. We can only find one example in the literature (also shown in Scheme 1, top) where ammonium carbonate NH_4_CO_3_ decomposes in an ethanolic solution of hydrated MgCl_2_ and the diyne 10,12-pentacosadiynoic acid (PCDA), to give [MgCO_3_]_*n*_ of 3–4 nm average size after being surrounded by a shell of PCDA (total particle size ≈ 6.3 nm).^8^ Despite the remarkability of this synthesis, the slow CO_2_ and NH_3_ release from NH_4_CO_3_ provokes the capture of the former by the Mg salt after HCl quenching, requiring 3 days to give a relatively big metal carbonate nanoparticle compared to the reported subnanometric [CaCO_3_]_*n*_ clusters. Furthermore, the surrounding PCDA shell should hamper any external coordination by the reactants during a catalytic procedure.^17^ In view of this, we considered the possibility of employing a simpler and faster TEA-mediated procedure to prepare the [MgCO_3_]_*n*_ clusters (see Scheme 1, bottom). The feasibility of this hypothesis is supported by previous studies, which show that (1) a controlled MgCl_2_ aggregation occurs in EtOH solutions,^18^ circumventing the higher affinity of Mg^2+^ for water compared to Ca^2+^,^19^ (2) the beneficial action for cluster formation of similar bases to TEA,^20,21^ (3) both [MgCO_3_]*_n_* and [CaCO_3_]_*n*_ clusters are implicated in the formation of the corresponding bulk carbonates, either after being detected^22–24^ or modeled,^25^ and (4) computational studies reveal the energetic stability of [MgCO_3_]_*n*_ (*n* up to 16) clusters.^26^ All of this literature encourages us to test the fast TEA methodology for the synthesis of [MgCO_3_]_*n*_ clusters and, if successful, to study the potential catalytic activity of the clusters in organic synthesis.

## Results and Discussion

### Synthesis and Characterization of the [MgCO_3_]_*n*_ Clusters

[MgCO_3_]_*n*_ clusters were obtained by dissolving MgCl_2_ (244 mg, 4.08 mmol) in ethanol (300 mL) at room temperature, assisted by sonication, to ensure complete solubilization. Upon addition of TEA (3.6 mL, 26 mmol) and subsequent bubbling of CO_2_ (3 bar) for 40 min, the solution gradually turned turbid, providing a first indication of cluster nucleation (pictured in Figure 1a). After an additional stirring period of 10–30 min, the resulting suspension was subjected to repeated centrifugation and ethanol washing to remove residual solvent and amines. The whitish solid thus isolated was dried and could be readily redispersed in minimal ethanol, yielding a stable colloidal dispersion of [MgCO_3_]_*n*_ clusters suitable for further characterization. To gain further insight into the chemical nature of the newly formed [MgCO_3_]_*n*_ clusters, we studied the different synthetic variables. These complementary experiments also allowed us to assess the robustness and versatility of the methodology. First, the synthesis was extended to different bases. It was observed that [MgCO_3_]_*n*_ clusters could also be obtained from ethanolic MgCl_2_ solutions when TEA was replaced with *N*,*N*-diisopropylethylamine (DIPEA), *N*-methylpyrrolidine (NMPy), 1,4-diazabicyclo[2.2.2]­octane (DABCO), or combinations thereof. This finding underscores the importance of an aliphatic tertiary amine in stabilizing a metastable precursor phase of the clusters through the formation of hydrogen bonds with protonated carbonate groups. Consistently, when aniline, pyrrolidine, or KOAc were employed, no turbidity was detected, thereby supporting the requirement of a relatively strong alkyl amine base. In parallel, the role of the reaction solvent was examined. Using MgCl_2_ as a precursor and TEA as a base, different polar solvents were tested, including methanol, isopropanol, water, and their mixtures. The use of water, either alone or in combination with alcohols, did not lead to the appearance of turbidity, which typically precedes cluster formation, as was also the case with methanol. In contrast, when ethanol was employed, turbidity was observed upon addition of the amine to the salt solution prior to the CO_2_ bubbling, and the distinct nature of the resulting solid was confirmed by FT-IR analysis. These results clearly indicated that the presence of ethanol was necessary for cluster formation. Furthermore, the use of ethanol as solvent with TEA confirmed that different Mg halides were suitable precursors for the synthesis of these materials. Details of these experiments, together with the basic characterization of the resulting [MgCO_3_]_*n*_ samples (FT-IR, EA, and DLS), are provided in Section 2.2 of the Supporting Information. To enrich the discussion of this work, it was considered appropriate to compare the nature of our newly obtained [MgCO_3_]_*n*_ material with that of its reported calcium analogue. [CaCO_3_]_*n*_ clusters were prepared following the protocol described in the literature (see supporting data for more details), and their characterization was fully consistent with the data reported therein. As we confirmed, the characterization results for [MgCO_3_]_*n*_ are closely aligned with those observed for [CaCO_3_]_*n*_, thereby ensuring a fair comparison between the two systems in subsequent catalytic studies. For clarity and to enable direct comparison, the following discussion will focus on the [MgCO_3_]_*n*_ clusters prepared under the initially described conditions.

**Figure 1:**
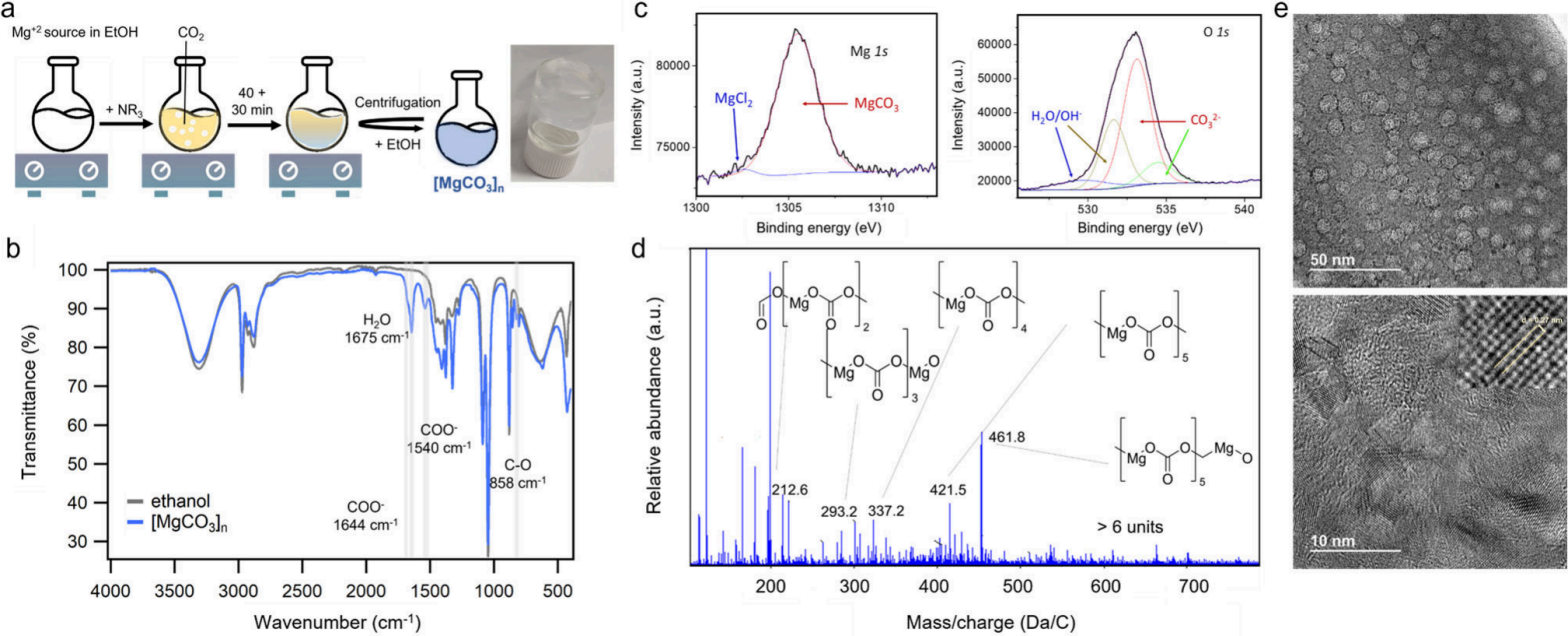
(a) Scheme of the synthetic procedure for preparing [MgCO_3_]_*n*_ clusters. (b) Attenuated total reflection Fourier transform infrared (FT-IR) spectrum of the (MgCO_3_)_*n*_ clusters (blue line). The spectrum of neat ethanol is used as a reference (gray line). The spectra show the peak corresponding to the carbonate groups in (MgCO_3_)_*n*_ and H_2_O (or N···H–O bonds between TEA and protonated carbonate). (c) Mg (left) and O (right) 1s XPS of the dried [MgCO_3_]_*n*_ clusters. (d) Matrix assisted laser desorption/ionization time-of-flight mass spectrum (MALDI-TOF MS) of the [MgCO_3_]_*n*_ clusters. (e) High resolution transmission electron microscopy (HR-TEM) images of the [MgCO_3_]_*n*_ clusters prepared with TEA, for different samples and at different magnifications. (inset) Crystallographic interplane distance for [MgCO_3_]_*n*_ of approximately 0.27 nm, which can be assigned to the (104) planes of rhombohedral MgCO_3_ (magnesite).

Figure 1b shows the attenuated total reflection Fourier transform infrared (FT-IR) spectrum of a MgCl_2_ ethanolic solution after bubbling CO_2_ in the presence of TEA, precipitating, and washing with neat EtOH (the final solution can also be in the form of dispersion at room temperature, depending on the final amount of clusters). New signals corresponding to the carbonate groups (two COO^–^ and one CO stretching bands at 1644, 1540, and 858 cm^–1^, respectively) appear, indicating the formation of carbonate species, in accordance with the FT-IR spectrum of bulk MgCO_3_ (Figure S2, top). Besides, a new band corresponding to either ammonium chloride or scissor bend H_2_O (NH or O–H at 1675 cm^–1^, as a shoulder band) also appears, supporting the quenching of released HCl. The FT-IR spectra obtained for the clusters synthesized using the other tertiary amine-derived bases (DIPEA, NMPy, and DABCO) exhibit an identical spectral pattern. The removal of volatiles under a vacuum gives a white solid that accounts for 7.1 ± 1 mg·mL^–1^ in the new ethanolic solution, in good accordance with the expected amount of MgCO_3_ to be formed after complete conversion of the starting anhydrous MgCl_2_. Remarkably, the same FT-IR spectrum is obtained after redispersion of the white solid in the same amount of EtOH and heating or not at 110 °C (Figure S2, bottom), showcasing the thermal stability of the MgCO_3_ material either as a dry gel or as a solution/dispersion in EtOH, and the reversibility of the solution/dispersion process. Inductively coupled plasma–optical emission spectroscopy (ICP-OES) measurements of this dry gel gave a 17.0 ± 1.2 Mg wt %, and the elemental analysis of this gel (Table S1) confirms that the C atom wt % in the new material accounts for the moles of Mg, to give a 98% yield of MgCO_3_ if ethanol is not considered. However, the EA reveals that the TEA species are very minor in comparison with the TEA species in [CaCO_3_]_*n*_ clusters (barely any N is detected for the former). Besides, the H atom wt % is much higher for MgCO_3_, in line with the higher tendency of MgCO_3_ to trap water (and perhaps EtOH) respect to CaCO_3_,^27^ suggesting that the MgCO_3_ species here are better stabilized by the water molecules present in the ethanolic solution rather than by TEA species. Similar results were obtained with DIPEA and NMPy. These experiments, carried out with amines of higher boiling points, allow us to exclude the possibility that the amine is simply lost during the rotary evaporation step. Altogether, the results strongly suggest that the [MgCO_3_]_*n*_ clusters are not stabilized by amines. In contrast, the [MgCO_3_]_*n*_-DABCO sample shows a higher N content than the previous ones (see entry 2 of Table S2), suggesting that the use of this cyclic diamine somewhat triggers the incorporation onto the clusters. In any case, the analyzed percentage of N would correspond to <0.1 molecules of DABCO per unit of MgCO_3_.

The Mg 1s X-ray photoelectron spectrum (XPS) of the dry MgCO_3_ gel is shown in Figure 1c, and, after deconvolution, it can be seen that >98% of the Mg signal belongs to Mg^2+^–O bonds and <2% to MgCl_2_ (see Figure S3 for the total XPS survey). These results exactly agree with the calculated yield of the reaction. The O 1s XPS spectrum, also shown in Figure 1c, confirms the presence of carbonates as major species together with water/hydroxyl moieties, in an ∼1.7:1 carbonate to water ratio. Besides, the C 1s XPS differentiates the signal corresponding to carbonate at 289.0 eV (Figure S3), and the minor presence of TEA is confirmed by the weak signal in the N 1s XPS (Figure S3), which accounts for <0.1% of the carbonate species. Ethanol species are not found here, since the extreme vacuum applied in the XPS pre-treatment chamber removes all the weakly adsorbed volatile species. ^1^H and ^13^C nuclear magnetic resonance (NMR) measurements of the gel redissolved in CD_3_OD show the presence of water and TEA in ∼1:2 and 1:100 mol % respect to the MgCO_3_ entities calculated by EA, respectively (Figure S4), and this amount of water was confirmed by a Karl–Fisher titration (100 parts-per-million of water in the MgCO_3_ ethanolic solution). Therefore, the combined EA, XPS, and NMR results suggest [MgCO_3_]_2–9_·2–6H_2_O (in average, [MgCO_3_]_5_·3H_2_O) as a potential molecular formula for our white dry gel. Figure 1d shows the matrix assisted laser desorption/ionization time-of-flight mass spectrum (MALDI-TOF MS) for the gel between 100 and 800 *m*/*z*, and the periodic appearance of masses compatible with hydrated [MgCO_3_]_*n*_ clusters between 2 and 9 units, peaking at 5 units. The extrapolation of the Gaussian curve give smaller oligomers up to 3–4 units. These results are in good agreement with the formula estimated from the combined EA/NMR/XPS analyses. These MgCO_3_ clusters will have a size between 0.6 and 2 nm (peaking at ∼1 nm for [MgCO_3_]_5_) and, in accordance, the corresponding dynamic light scattering (DLS) measurements of the ethanol solution showed a wide peak centered at ∼1.3 nm (DLS size resolution is >1 nm, Table S3 and Figure S5). The sample prepared with DIPEA exhibits a comparable DLS value; however, the samples synthesized using cyclic amines such as NMPy and DABCO display higher values. Nevertheless, all of them remain significantly below the microscopic particle size observed for bulk MgCO_3_ (∼1500 nm). A clear correlation was found between the cluster size and the basicity of the employed base, demonstrating that the degree of cross-linking in (MgCO_3_)_*n*_ oligomers can be effectively tuned by modulating the base strength (Table S3). As expected, stronger bases act as more efficient capping agents (but removable), leading to the stabilization of smaller oligomeric species.

The powder X-ray diffractograms (PXRD) of [MgCO_3_]_5_·3H_2_O dry gel did not show any diffraction peak (size resolution >2 nm), in contrast to bulk MgCO_3_ and starting MgCl_2_ (Figure S6, top), and this result is in accordance with the conclusion that the higher MgCO_3_ clusters obtained are <2 nm. The same result is observed for the rest of the prepared clusters (Figure S6, bottom). Together, these results strongly support the synthesis of [MgCO_3_]_*n*_ clusters with the formula [MgCO_3_]_2–9_·H_2_O_2–6_, many of them subnanometric. The stabilization of ultrasmall MgCO_3_ entities by water is in accordance with the literature (see ahead).^28^ The isolation of this new subnanometer form of [MgCO_3_]_*n*_ enables to explore the impact of size reduction on the physicochemical properties of the material, which, as will be discussed later, are highly relevant to its catalytic performance. For instance, pH measurements in water revealed that the dried [MgCO_3_]_*n*_ sample exhibits a basic character significantly higher (by 2 units) than that of its bulk analogue (see Figure 1f and Table S3). A similar trend was also observed for the [CaCO_3_]*_n_* clusters.

Figure 1e shows two representative high resolution transmission electron microscopy (HR-TEM) images of the [MgCO_3_]*_n_* clusters prepared with TEA (more images can be found in Figure S7). The results show the presence of aggregates of ∼10 nm, composed of crystalline nanoclusters of ∼1–2 nm, exposing the typical crystallographic interplanar distance for (104) planes of rhombohedral MgCO_3_ (magnesite) (∼0.27 nm) in different orientations. In contrast, the MgCO_3_ nanoparticles prepared with DABCO showed a much higher particle size (∼100 nm) with only one crystalline orientation at higher magnification views, in accordance with the higher particle size (Figure S8). These results are in good accordance with previous characterization. To further discard that a slow crystallization on the TEM grid is triggering a MgCO_3_ nanocrystal formation, the same slow crystallization for the TEA-mediated MgCO_3_ nanocluster sample was performed on a PXRD cell, and measured, without any peak corresponding to crystalline planes (Figure S9). This result confirms that the lack of signals in the PXRD technique comes from the ultrasmall size (<2 nm) of the MgCO_3_ nanocrystals.

### Structural Modeling and Comparison of the CaCO_3_ and MgCO_3_ Clusters

Density functional theory (DFT) was then implemented to mimic the interaction of both CaCO_3_ and MgCO_3_ clusters with the surrounding molecules. At an early stage, two model systems have been built up by using eight metal–carbonate units, which lead to complete [CaCO_3_]_8_ and [MgCO_3_]_8_ entities. The initial geometries correspond to previously reported global minimum structures by Dixon and co-workers,^26^ which were further optimized at the selected level of theory. The nature of the real minima in the potential energy surface is further confirmed by computing the associated normal modes (in the absence of imaginary frequencies). The surfaces of the clusters were subsequently modified with one water molecule, which is allowed to search for the most favorable anchoring site, and the same procedure was applied for one molecule of TEA (Figure S10, the final optimization was conducted prior to the computing complexation energies).^29^

The simulations performed are summarized in Figure 2. As illustrated, the H_2_O molecules are prone to coordination by oxygen–metal interactions rather than through hydrogen bonds via carbonate anions in both clusters. In contrast, the computed structures with TEA are characterized by metal coordination with the amine nitrogen atom in both cases. A close inspection reveals significant dissimilarities in the predicted complexation energies (Δ*E*, in kcal/mol). As one can see, the interaction of the Mg-based cluster with water (Δ*E* = −37 kcal/mol) is 10 kcal/mol larger (more negative) than the value computed for its Ca counterpart (Δ*E* = −27 kcal/mol). This numeric outcome matches the observed tendency of [MgCO_3_]_*n*_ clusters to trap water. More striking values are obtained upon TEA decoration. The interaction energy for the [CaCO_3_]_8_–TEA complex (Δ*E* = −39 kcal/mol) is 12 kcal/mol more stable than the value computed with water, in agreement with the observed preference of [CaCO_3_]_*n*_ for TEA. On the contrary, both [MgCO_3_]_8_–TEA and [MgCO_3_]_8_–H_2_O complexes lead to a very similar energy. This result points to a competition between TEA and water for occupying sites in the [MgCO_3_]_8_ surface, which in turn explains the minor presence of TEA compared to [CaCO_3_]_8_.

**Figure 2:**
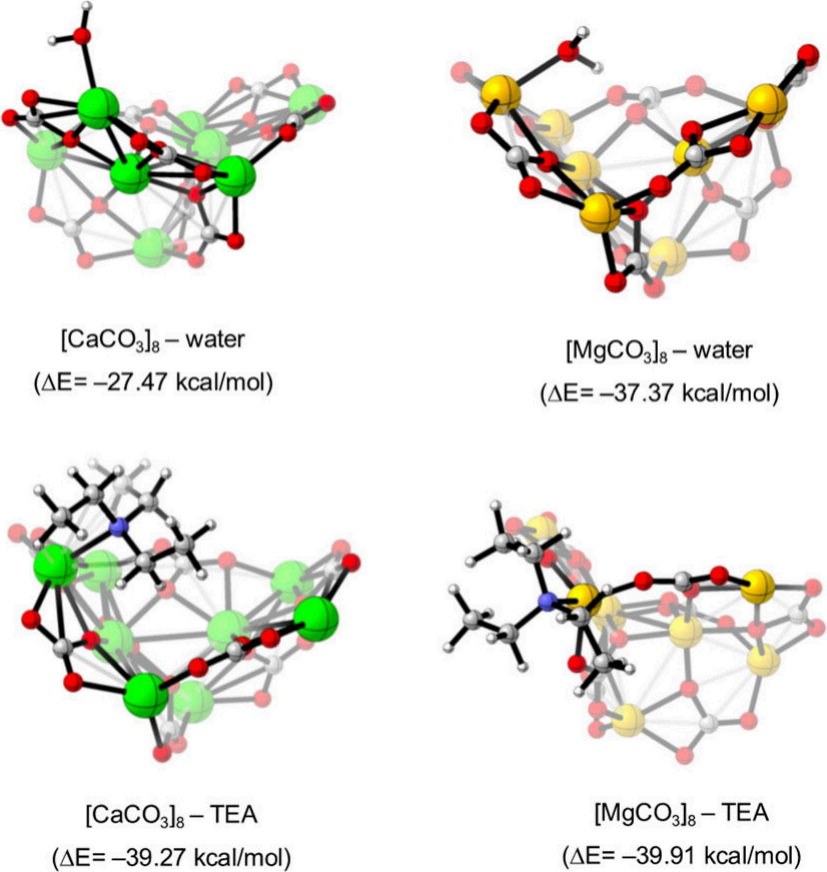
Optimized cluster models after interaction with one water molecule (top) and one TEA molecule (bottom panel). Computed complexation energies are given in parentheses. Color atom code: green, Ca; orange, Mg; white, H; gray, C; red, O; blue, N.

Besides, the zeta potential of the ethanol solution of the hydrated [MgCO_3_]_*n*_ clusters is +41 mV, which indicates good stability and confirms that the carbonate units in the cluster face toward the outer shell. This effect is also consistent with the increase in pH observed for the nanometric carbonate form. FT-IR measurements confirm the stability of the [MgCO_3_]_*n*_ clusters in water (Figure S11, top). Indeed, the [MgCO_3_]_*n*_ clusters precipitate much more rapidly than bulk MgCO_3_ (Figure S11, bottom). It must be remarked here that both MgCO_3_ materials (clusters and bulk) are insoluble, but the [MgCO_3_]_*n*_ clusters are more basic and insoluble, enabling new unforeseen applications in water.

### Catalysis with CaCO_3_ and MgCO_3_ Clusters

In light of the basic properties exhibited by the clusters and considering previous reports in which carbonates have been employed as catalysts, our catalytic studies focused on nucleophilic alcohol addition reactions. The first reaction studied was the transesterification of dimethyl carbonate **1** (DMC) with ethanol. This reaction makes use of bio-based compounds, and the organic carbonate products ethyl methyl carbonate (EMC) **2** and diethyl carbonate (DEC) **3** are considered essential components for pharmaceutical,^30,31^ agrochemicals,^32^ polymers,^33^ lubricants,^34^ coatings,^35^ varnish,^36^ and building blocks for chemical reactions.^37–39^ In particular, EMC **2** shows a high flash point, low volatility and low toxicity,^40,41^ and it is a promising electrolyte for lithium-ion batteries^42^ and blender for gasoline.^43–45^ Therefore, the selective nucleophilic addition of EtOH to DMC **1** is of potential industrial interest.

The catalytic results in Table 1 show that both the [MgCO_3_]_*n*_ clusters and bulk MgCO_3_, in 1.5 mol % amount respect to DMC **1**, show some catalytic activity for the reaction at 40 °C, above the blank and the corresponding CaCO_3_ materials [five times more according to gas chromatographic (GC) analyses, entries 1–5]. This enhanced catalytic activity for the [MgCO_3_]_*n*_ clusters is confirmed after increasing the temperature to 80 °C, achieving a 55% conversion of **1** with the [MgCO_3_]_*n*_ clusters but only a 14% with the [CaCO_3_]_*n*_ clusters, under identical reaction conditions (entries 6–7). Kinetic experiments confirm the higher initial rate of the [MgCO_3_]_*n*_ clusters (Figure S12). In all these experiments, EMC **2** was the major product with respect to DEC **3**, as expected at low to moderate conversions of **1**. Gratifyingly, a nearly complete conversion of **1** (98%) was obtained with the same amount of [MgCO_3_]_*n*_ clusters at 110 °C, while the bulk MgCO_3_ catalyst just achieved a 40% conversion (entries 9–10, see kinetics in Figure S13). It must be recalled here that the [MgCO_3_]_*n*_ clusters are stable at 110 °C (see Figure S2). The selectivity for **2** and **3** was approximately 1:1 at the highest conversion. Please notice that the uncatalyzed reaction does not proceed yet at this temperature (entry 8). The [CaCO_3_]_*n*_ clusters and bulk materials gave a 70% and 8% conversion at 110 °C, respectively (entries 11–12), a significantly lower catalytic activity than the MgCO_3_ materials, in line with the results at lower temperatures (see kinetics in Figure S14). Thus, at this point, we decided to decrease the amount of the MgCO_3_ materials to 0.6 mol %, to give a 96% conversion of **1** with the clusters while 21% with the bulk material (entries 13–14, see kinetics in Figure S15). As can be seen in Figure 3a, the initial rates at different catalyst concentrations strongly indicate that the carbonate materials as soluble clusters are much more catalytically active for the transesterification of **1** with EtOH than the corresponding bulk counterparts^46^ and that the MgCO_3_ materials are, at least, more active than the CaCO_3_ materials (typically 2–3 times more active in both cases, see the comparison in initial rates and turnover numbers in Table S4 and Figure S16). An initial turnover frequency (TOF_0_) of 210 h^–1^ is obtained when referred to an averaged carbonate cluster with five units (Figure 3b). The higher catalytic activity of MgCO_3_ with respect to CaCO_3_ supports our starting hypothesis on the convenience of a harder (more ionic) carbonate to activate the carbonyl bonds, making sense to the synthesis of the [MgCO_3_]_*n*_ clusters. To gain further insight into the origin of the main differences between the nanocluster-based material and the bulk counterpart, we performed carbon dioxide temperature-programmed desorption (CO_2_-TPD) experiments on both samples. The results reveal significant differences in the nature of the basic sites between the [MgCO_3_]_*n*_ dried clusters and bulk MgCO_3_ (see Figure S17). The bulk MgCO_3_ sample exhibits CO_2_ desorption at higher temperatures and over a broader range, indicating the presence of more heterogeneous basic sites. This behavior can be attributed to well-coordinated carbonate species within the extended crystalline lattice of bulk MgCO_3_, which stabilize adsorbed CO_2_ more effectively through strong electrostatic interactions. In contrast, the nanometric [MgCO_3_]_*n*_ sample displays a narrower desorption peak centered at slightly lower temperatures, consistent with a more uniform population of basic sites of moderate strength. These sites are associated with less-coordinated surface carbonate species, which are more susceptible to hydration and structural distortion on the nanoscale. Such surface environments result in CO_2_ adsorption that is weaker but more homogeneous compared to the bulk material. These differences highlight the distinct surface chemistry arising from variations in particle size and structural organization, and they rationalize the observed divergence in catalytic performance between materials. Therefore, these results suggest that the catalytic activity of the material is not governed by its overall basicity but rather by the availability of basic sites, which are more accessible in the cluster-based structures.

**Table 1:**
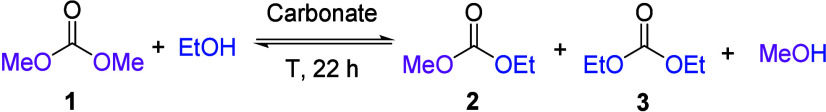
Catalytic Results with [MgCO_3_]_2–9_·H_2_O_2–6_ and [CaCO_3_]_4–13_·TEA_0.1_ Clusters, and the Bulk Counterparts, for the Transesterification Reaction of DMC **1** (1 mmol) with Ethanol (0.5 M)^a^

				selectivity (%)	
entry	catalyst	*T* (°C)	conv. DMC **1** (%)	EMC **2**	DEC **3**
1	none	40	<1	>99	–
2	bulk MgCO_3_	40	<5	>99	–
3	[MgCO_3_]_*n*_	40	<5	>99	–
4	bulk CaCO_3_	40	<1	>99	–
5	[CaCO_3_]_*n*_	40	<1	>99	–
6	[MgCO_3_]_*n*_	80	55	92	8
7	[CaCO_3_]_*n*_	80	14	98	2
8	none	110	<1	>99	–
9	[MgCO_3_]_*n*_	110	98	50	50
10	bulk MgCO_3_	110	40	95	5
**11**	**[CaCO**_**3**_ **]**_***n***_	**110**	**70**	**86**	**14**
12	bulk CaCO_3_	110	<8	>99	–
**13** ^a^	**[MgCO**_**3**_ **]**	**110**	**96**	**51**	**49**
14^a^	bulk MgCO_3_	110	21	95	5

a Catalyzed by different catalyst amounts and at different temperatures (40–80–110 °C) after 22 h of reaction. Reactions were performed by duplicate. GC results.

b 0.6 mol %.

**Figure 3:**
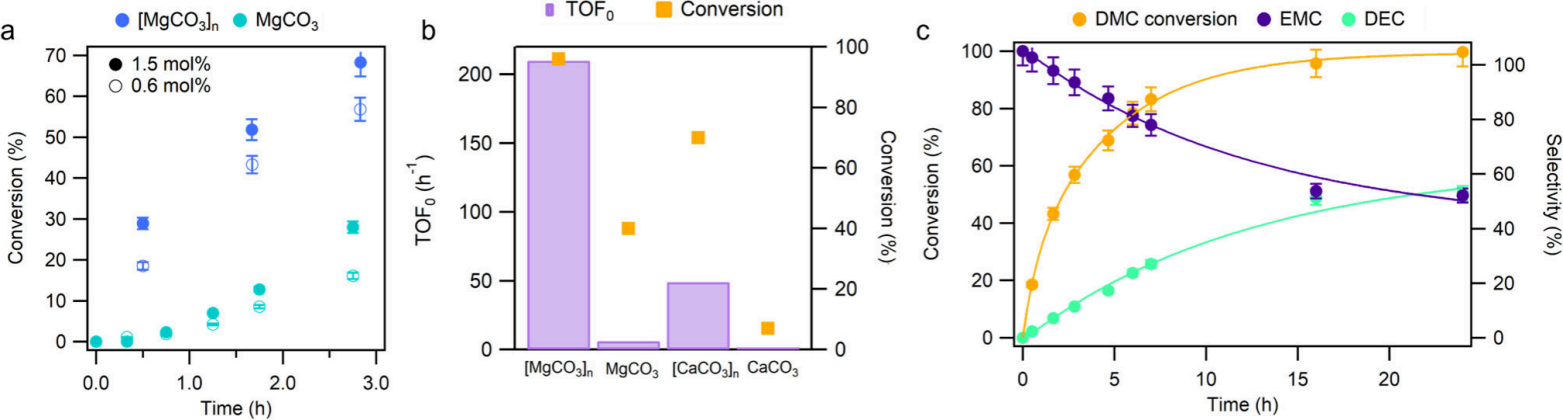
(a) Comparison of the kinetics profiles until 3 h reation time for bulk MgCO_3_ and the [MgCO_3_]_*n*_ clusters at 110 °C, with different quantities of catalyst. GC results. Error bars account for 5% uncertainty. (b) Initial turnover frequency (TOF_0_) of magnesium and calcium carbonates clusters and bulk form. (c) Kinetic profile for the transesterification reaction of DMC **1** with ethanol catalyzed by [MgCO_3_]_2–9_·H_2_O_2–6_ (0.6 mol %) at 110 °C. GC results. Error bars account for a 5% uncertainty.

Figure 3c shows the kinetic profile for the transesterification reaction of DMC **1** in EtOH with the [MgCO_3_]_*n*_ clusters as a catalyst, where the experimental data are well fitted by a first-order exponential function, indicating apparent first-order kinetic behavior without any induction period. Remarkably, the kinetic curve with the [MgCO_3_]_*n*_ clusters after being dried to gel and dissolved back into the EtOH solution was nearly the same, confirming the stability of the clusters after drying and redissolution/redispersion. Besides, the [MgCO_3_]_*n*_ clusters dispersed in water, recovered, and dried gave similar catalytic results (82% conversion with 73% selectivity to EMC **2**), and the reaction could be scaled-up to gram scale without any change neither in the conversion nor selectivity to the EMC **2** product (96% conversion with 64% selectivity to EMC **2**). The recyclability experiments indicate that both the catalytic activity and the selectivity toward EMC are maintained over two consecutive reuse cycles (Figure S18). However, a decrease in activity is observed during the third reuse. This decrease is likely attributed not to intrinsic deactivation of the [MgCO_3_]*_n_* clusters but rather to the progressive loss of catalyst mass during handling. Since the clusters are present in the form of an ethanolic gel, complete recovery of the catalyst after each cycle is challenging (Figure S19), leading to a gradual reduction in the effective amount of catalyst, which correlates with the observed decrease in activity. Overall, these results suggest that the clusters themselves remain stable under the reaction conditions and the loss in activity over successive cycles is primarily due to mechanical handling limitations rather than chemical degradation. In addition to these experiments, the potential interference or involvement of amines present in the cluster was also investigated. Considering the proposed structure of the Ca clusters, i.e., [CaCO_3_]_4–13_·TEA_0.1_, where TEA is present as a quaternary amine, we conducted an additional experiment under optimized reaction conditions with one equivalent of trimethylammonium chloride (TMACl), and any catalytic activity was not observed after 22 h of reaction. Even the use of 10 equiv of TMACl (0.0173 mmol) did not give any product (Scheme S1), and its combination with bulk CaCO_3_ (1.42 mg, 0.014 mmol) only yielded the original catalytic activity of the latter (<10% conversion). These results exclude any hiding catalytic activity by the stabilizing molecules of TEA beyond the studied carbonates. Additionally, the catalytic activity of the remaining amine-based clusters was also investigated. First, the transesterification reaction of DMC **1** was carried out at 110 °C using 0.6 mol % of catalyst. Similar to the behavior observed for the [MgCO_3_]_*n*_–TEA sample, all other clusters led to a complete conversion. To more accurately evaluate potential differences in their catalytic performance, the catalyst loading was decreased to 0.45 mol %, where only the [MgCO_3_]_*n*_–DABCO sample achieved full conversion, likely due to the higher content of DABCO molecules in the gel. In fact, when compared to the TEA-based clusters, the concentration of DABCO is estimated to be about 8-fold greater. It is observed that the catalytic behaviors of the samples prepared with TEA (1.3 nm) and DIPEA (1.5 nm) are very similar, whereas lower conversions are obtained with NMPy (13.5 nm). This finding may suggest an influence of particle size and the number of exposed active sites on the overall catalytic activity (Figure S20).

The scope of different alcohol nucleophilic reactions catalyzed by the [MgCO_3_]_2–9_·H_2_O_2–6_ clusters was then studied. Figure 4 shows the results not only for carbonates but also for esters with EtOH and MeOH as nucleophiles. The latter was possible after drying the [MgCO_3_]_*n*_ clusters and redissolving the white gel in MeOH instead than in EtOH, and FT-IR measurements confirmed the stability of the clusters in MeOH (Figure S21). The first carbonate studied (apart from **1**) was ethylene carbonate (EC) **4**. This carbonate is part of the OMEGA industrial process [“only monoethyl glycol (MEG) advantage”],^47^ which produces ethylene glycol from ethylene through the hydrolysis of intermediate **4**,^48^ avoiding the direct hydrolysis route with the hazardous reactant ethylene oxide.^49^ The industrial OMEGA synthesis employs highly acidic catalysts, however, the implementation of a more practical and environmentally benign catalyst such as the [MgCO_3_]_*n*_ clusters would be of interest.^50^ The result shows that the methanolysis of **4** occurs in the presence of the clusters (1.5 mol %) in 99% yield, thus giving ethylene glycol **5** in very high yield. An equally high yield (96%) was achieved after the ethanolysis of **4**. Furthermore, the ethanolysis of propylene carbonate (PC) **6** also occurs in very high yields with either MeOH (85%) or EtOH (98%), to give the desired propylene glycol **7** in nearly quantitative yield. It is noteworthy to comment here that the different reactivity of MeOH and EtOH comes from a subtle balance between relative nucleophilicity, leaving group rate, and volatility: while MeOH is more nucleophilic than EtOH, it is, at the same time, a better nucleofuge and more volatile, remaining for less time in the liquid phase at the optimized reaction temperature (110 °C). Therefore, it is difficult to predict when MeOH or EtOH would be a more effective alcoholysis agent under our reaction conditions. For instance, the transesterification reaction of DEC **3** with MeOH to give EMC **2** and DMC **1**, i.e., the reverse reaction to that studied for reaction optimization (see Table 1 above), gave a 45% conversion, much lower than the ethanolysis of DMC **1** (98%, entry 8 in Table 1). However, both the ethanolysis of dimethyl malonate **8** and the methanolysis of diethyl malonate **10** gave excellent conversions (98–99%) and selectivity to the symmetric diesters (72–82%). To assess which one, EtOH or MeOH, was the reactant of choice for a more efficient alcoholysis reaction, the reactions were scaled five times (on a basis from 0.25 to 1.25 mmol of substrate) for both carbonates and malonates, and kinetic experiments were carried out. In this way, we expected to have a more accurate picture of the reaction behavior at reasonable scales through the initial reaction rates. The results show that, for carbonates, the scaling-up preserves well the reaction yields and that the nucleophilic attack of EtOH on the methyl carbonate **1** is 5 times faster than the reverse reactivity (Figure S22). For malonates, the scaling-up also preserves the high product yields, and the reactivity trend was the same than for carbonates, i.e., EtOH 5 times faster than MeOH (Figure S23). Thus, we decided to use preferentially EtOH as a nucleophile in the following reactions.

**Figure 4:**
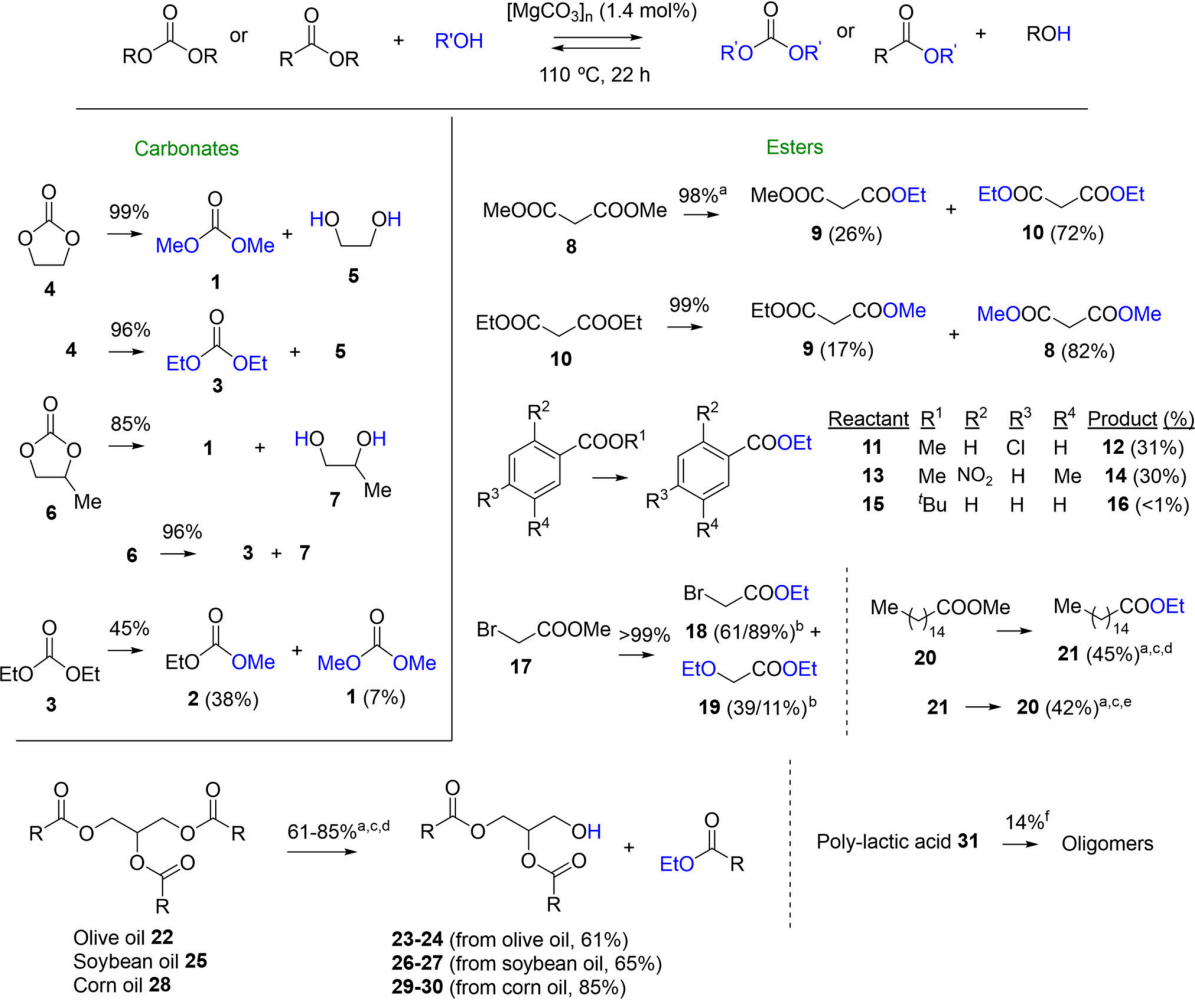
Scope for different alcohol nucleophilic reactions catalyzed by [MgCO_3_]_2–9_·H_2_O_2–6_ (1.5 mol %) at 110 °C for 22 h. The reacting solutions are at 0.5 M concentration in the substrate. Reactions performed by duplicate. Combined NMR and GC results after weighing the resulting crude product. ^a^46 h reaction time. ^b^The second result is for 0.6 mol % catalyst. ^c^5 mol % catalyst. ^d^170 °C reaction temperature. ^e^150 °C reaction temperature. ^f^THF as a co-solvent.

The quantitative transesterification of malonates **8** and **10** must be remarked since these molecules have a similar reactive site between the two ester activating groups, with acidic hydrogens.^51^ Thus, we tested the ethanolysis of different methyl benzoates; however, they were not as effective as malonates. The 4-chloro derivative **11** gave a 31% yield of the corresponding ethyl benzoate **12**, as assessed not only by GC but also by ^1^H, ^13^C, and distortionless enhancement by polarization transfer (DEPT) NMR analyses (Figure S24). The polysubstituted methyl benzoate **13** gave a similar yield of the corresponding ethyl benzoate **14** (30%), while *tert*-butyl benzoate **15** was completely unreactive, and methyl benzoate **16** was not detected. When an equimolecular mixture of dimethyl malonate **8** and *p*-chloromethylbenzoate **11** was tested as substrates, only **8** exclusively reacted with 96% conversion (Figure S25), confirming the high selectivity of the [MgCO_3_]*_n_* cluster catalyst for alkyl rather than aromatic esters. In contrast, bromo methyl acetate **17** reacted completely to give the corresponding ethyl ester **18** in very high yield after decreasing the amount of catalyst since, otherwise, the (bis)­ethoxy-substituted product **19** was also formed (see Figure S26 for the corresponding GC, ^1^H, ^13^C, and DEPT NMR analyses). This result indicates that nucleophilic substitutions of halogens are also possible in the presence of the [MgCO_3_]_*n*_ clusters.^52^

The transesterification of methyl palmitate **20** was then attempted, since the ethanolysis of bio-esters is of industrial interest.^53^ The transformation of methyl palmitate **20** to ethyl palmitate **21** occurred in 30% yield at 110 °C and in 45% yield at 170 °C, after 46 h of reaction, which indicates that the bio-ester can be trans-esterified with the [MgCO_3_]_*n*_ clusters catalyst. The reverse reaction, i.e., the methanolysis of ethyl palmitate **21** to methyl palmitate **20**, occurs in similar yields (42%). With these results in hand, we speculated with the possibility that the ethanolysis of fatty esters may occur under the catalysis of [MgCO_3_]_*n*_ clusters and, in accordance with our hypothesis, a commercial sample of olive oil **22** hydrolyzed to **23** and formed the corresponding trans-esterified ethyl ester **24** in 61% yield, while soybean oil **25** gave a 65% yield (Figures S27–S28) and corn oil **28** gave a remarkable 85% yield, after quantification by NMR (Figure S29). Although a single hydrolysis is indicated in Figure 4, a double hydrolysis could occur at this high conversion. These results indicate that complex fatty ester mixtures can be broken under the catalytic action of the [MgCO_3_]_*n*_ clusters, under milder reaction conditions than the current ones with extremely corrosive catalysts such as NaOH or KOH.^53,54^ Poly-lactic acid **31** was then employed as a substrate, since the transesterification/hydrolysis of **31** is of much interest for recycling of used polymers,^55,56^ and 14% degradation was obtained according to combined GC-MS and NMR quantification (Figure S30, see the new peaks at 170–176 ppm in the ^13^C NMR spectrum compared to the original peak at 177 ppm for polylactic acid **31**). Although the conversion of **31** is still low, the benignity of the [MgCO_3_]_*n*_ clusters compared to strong bases or acids commonly employed as catalysts for this reaction makes the system worthy of further studying, moreover considering that, here, tetrahydrofuran (THF) had to be used as a cosolvent and the reaction temperature was moderate (110 °C).

## Conclusion

Ultrasmall soluble MgCO_3_ clusters (0.6 to <2 nm) have been prepared by the capture of CO_2_ into an ethanolic solution of MgCl_2_ in the same way as CaCO_3_ clusters of similar size^12^ but with a variety of amines and Mg sources. The MgCO_3_ clusters can be described by an average formula [MgCO_3_]_5_·3H_2_O, while the CaCO_3_ clusters can be described by an average [CaCO_3_]_8_·TEA_0.1_ formula. Thus, the main difference between both clusters is that MgCO_3_ is stabilized with water molecules while CaCO_3_ requires TEA molecules. Both materials showed an enhanced catalytic activity with respect to the bulk carbonates (>5 times) for different nucleophilic additions of the EtOH solvent, which can be replaced by MeOH after a drying/redissolving procedure. The exploration of different synthetic conditions for these materials has led to the conclusion that the synthesis is versatile and can be extended to the use of other tertiary amines, yielding ultrasmall MgCO_3_ clusters that are potentially active in catalysis. The MgCO_3_ clusters catalyze transesterification reactions of carbonates and esters in good yields, including current industrial reactions and bio-based materials. Since the only byproduct formed during the synthesis of the clusters is the HCl captured by the amine (MgCl_2_ + CO_2_ + H_2_O + 2NR_3_ → MgCO_3_ + 2HCl·NR_3_), the final protonated amine can be recycled as amine + MgCl_2_ after treatment with a Mg base such as MgOEt (to also generate the alcohol solvent), providing a circular system. These results, overall, open the way to prepare subnanometric carbonate clusters rather than CaCO_3_ by a simple CO_2_ capture, of relevance in the current context where CO_2_ transformations are mandatory.^57^ The employment of these clusters as catalysts in organic synthesis^58–62^ and, perhaps, the understanding of carbonates pre-nucleation,^63–66^ would also benefit of the studies presented here.

## Experimental Section

### Synthesis of the [MgCO_3_]*_n_* Clusters

In a 500 mL two-neck flask, 244 mg of MgCl_2_ (or MgI_2_) (4.08 mmol) was added and dissolved in 300 mL of synthesis-grade ethanol at room temperature, using an ultrasonic bath to ensure that all the salt was fully dissolved. Once the salt was dissolved, stirring was maintained, and base (TEA; DIPEA, NMPy, DABCO, aniline, pyrrolidine, or KOAc) (26 mmol) was added. Subsequently, a stream of CO_2_ was bubbled through a CO_2_ cylinder (3 bar). Bubbling was maintained for 30 min. During this time, turbidity appeared, indicating the formation of the cluster. After 30 min, the bubbling was stopped, and the solution/dispersion was left stirring for an additional 30 min. After this time, the mixture was collected into centrifuge tubes and centrifuged to remove the solvent and amine residues. The whitish supernatant was washed with ethanol and centrifuged again. This process was repeated four times. Once the supernatant was clean, the residue was redispersed in the minimum amount of ethanol needed to prevent the cluster from settling at the bottom.

### Typical Reaction Procedure [Transesterification Reaction of Dimethyl Carbonate (DMC, **1**) with Ethanol]

DMC (84 μL, 1 mmol), the ethanolic solution of carbonate clusters (1.5 mol %), and additional EtOH reaching 2 mL (34.3 mmol) were added into a 10 mL glass vial equipped with a magnetic stir. The vial was sealed, and the resulting mixture was magnetically stirred over a temperature ranging from 40 to 110 °C on a heating plate for 22 h. The progress of the reaction was monitored by taking aliquots (25 mL) and analyzing them by gas chromatography (GC) after dissolving in 1 mL of diethyl ether, filtering through a 25 μm polyamide filter, and adding *n*-dodecane (5.5 mL, 0.025 mmol) as an external standard. The conversion and yield were obtained from calibration curves. At the end of the reaction, the volatiles were removed under rotavapory vacuum suction, the remaining crude was redissolved in CDCl_3_, the solids were filtered off, and the resulting product was weighted and analyzed by NMR.

## Supplementary Material



## Notes

Experimental section, compound characterization, and additional comments and references involving Tables S1–S4, Scheme S1, and Figures S1–S26 (PDF)
