# Supplementary material for: Ligand-Free MgCO3 Nanoclusters Catalyze Nucleophilic Alcohol Addition Reactions
Source: ACS Appl Mater Interfaces. 2026 Feb 10;18(7):11273–83. doi: 10.1021/acsami.5c21329 (PMC12954672; doi:10.1021/acsami.5c21329)
Supplement: Supplementary file 1 [file am5c21329_si_001.pdf]

Supporting Information

Ligand-free $MgCO_3$ nanoclusters catalyze nucleophilic alcohol addition reactions

Lluís Martínez-Belenguer,[a] Kateřina Zítová,[b] Jose Pedro Cerón-Carrasco,[c] Belén Lerma-Berlanga,[*,a] and Antonio Leyva-Pérez.[*,a]

[a] Instituto de Tecnología Química. Universitat Politècnica de València-Agencia Estatal Consejo Superior de Investigaciones Científicas. Avda. de los Naranjos s/n, 46022, Valencia, Spain. Phone: +34963877800; Fax: +349638 77809.

[b] Department of Organic Technology, University of Chemistry and Technology Prague, Technická 5, 16628, Prague 6, Czech Republic

[c] Centro Universitario de la Defensa, Academia General del Aire, Universidad Politécnica de Cartagena, C/ Coronel López Peña S/N, Santiago de La Ribera, 30720 Murcia, Spain.

Corresponding author: blerber@itq.upv.es and anleyva@itq.upv.es

Table of content

1. Experimental Section 3

General 3

Physical techniques 3

2. Synthesis and characterization of the materials 4

2.1. General synthetic procedure 4

2.2.Exploring the synthetic variables 6

3. Computational modelling and comparison of the Ca^{+2} and Mg^{+2} carbonate clusters 18

3.1. Computational details 18

3.2. Additional experiments 20

4. Catalytic reactions 21

4.1. Kinetics results 22

4.2. Reactive experiments 32

4.3. NMR characterization 36

References 45

1. Experimental Section

General

Reagents and solvents were obtained from commercial sources and were used without further purification otherwise indicated. Glassware was dried in an oven at 175 ºC before use.

Physical techniques

- Fourier transformed infrared (FT-IR) spectroscopy: Spectra were recorded on attenuated total reflection infrared spectroscopy, from 400 to 4000 cm^{-1}, by dropping a small sample on the ATR crystal. Due to the high ethanol content in the gels, the spectra of both the material and the solvent were superimposed to allow for clearer identification of the characteristic bands of the carbonate gel.

- X-Ray diffraction: Spectra were recorded in a CubiX PRO (PAN Analytical) spectrometer, with a Cu K(α) radiation source, 1.5406 Å wavelength.

- X-ray photoelectron spectroscopy (XPS): Measurements were performed on a SPECS spectrometer equipped with a Phoibos 150 MCD-9 analyser using a non-monochromatic Mg KR (1253.6 eV) X-ray source working at 50 W. The C1s peak has been set at 284.5 eV as the internal reference for the peak positions in the XPS spectra.

- Mass spectrometer with MALDI ionization source and time-of-flight analyzer (MS MALDI TOF): Samples were analyzed in a 5800 MALDI TOFTOF (ABSciex) in reflector positive mode, in a range 500 – 2000 m/z, at 2800 – 3500 of laser intensity.

- Elemental analysis: the sample was analyzed in a CHNS analyzer after thermally oxidizing the sample and quantitatively measuring the volatiles in a GC chromatograph. It should be noted that, due to the limitations of this technique, the gel sample must be measured in the solid state, which requires ethanol removal. This process is conducted under vacuum using a rotary evaporator. It is important to note that both gels, $(CaCO_3)_n$ and $(MgCO_3)_n$, were treated in the same manner, making the results directly comparable.

- Inductively coupled plasma-optical emission spectroscopy (ICP-OES) was determined by a Thermo Scientific ICAP Pro. The dry gel was disaggregated in concentrated HCl and later diluted in water before analysis.

- Nuclear magnetic resonance (NMR): NMR spectra were run in a Bruker Avance 300 DPX spectrometer (300 MHz for ^1H and 75 MHz for ^{13}C NMR) or in a Bruker Avance 400 spectrometer (400 MHz for ^1H and 101 MHz for ^{13}C NMR). Samples were dissolved in deuterated solvents, using the residual non-deuterated solvent as an internal standard (δ 7.26 for ^1H NMR and δ 77.16 for ^{13}C NMR in the case of $CDCl_3$). The carbon multiplicity was determined by DEPT experiments.

- Ultrahigh pressure liquid chromatography–electrospray ionization-time of flight-mass spectrum (UPLC–ESI-TOF-MS): The fragmentation pattern of the calcium carbonate chains was obtained with un UPLC-MS instrument, by directly infusing the liquid sample into the electrospray ionization chamber. The mass spectrometry was performed with a time-of-flight detector.

- Gas chromatography-mass spectrometry (GC-MS): Gas chromatographic analyses were performed in an instrument equipped with a 25 cm capillary column of 5% phenylmethylsilicone. n-dodecane was used as an external standard. GC-MS analyses were performed on a spectrometer equipped with the same column as the GC and operated under the same conditions.

- High-resolution TEM (HRTEM) analyses were performed on a JEOL JEM-2100F Field Emission Transmission Electron Microscope operating at 200 kV, after supporting the sample dissolved in ethanol on a grid and leaving to evaporate for 2 h.

- Carbon dioxide temperature-programmed desorption (CO_2-TPD) experiments were performed using a Quantachrome ChemStarWin Automated Chemisorption Analyzer to investigate the basic properties of the samples. Prior to analysis, the samples were pretreated under a flow of helium to remove physisorbed species. The samples were then exposed to 100% CO_2 (analytical grade) at a flow rate of 10.0 sccm, allowing CO_2 adsorption on the basic sites. After the adsorption step, the system was purged with helium to remove excess and weakly physisorbed CO_2. Temperature-programmed desorption was subsequently carried out under a continuous flow of helium as carrier gas, while the temperature was increased from 35 to 450 °C at a controlled heating rate. The desorbed CO_2 was continuously monitored using a thermal conductivity detector (TCD) coupled to a mass spectrometer, and the resulting desorption profiles were used to evaluate the strength and distribution of basic sites on the samples.

2. Synthesis and characterization of the materials

2.1. General synthetic procedure

- Synthesis of the $[MgCO_3]_n$ clusters: In a 500 mL two-neck flask, 244 mg of $MgCl_2$ (or MgI_2) (4.08 mmol,) are added and dissolved in 300 mL of synthesis-grade ethanol at room temperature, using an ultrasonic bath to ensure that all the salt is fully dissolved. Once the salt is dissolved, stirring is maintained, and base (TEA; DIPEA, NMPy, DABCO, aniline, pyrrolidine or KOAc) (26 mmol) is added. Subsequently, a stream of CO_2 is bubbled through using a CO_2 cylinder (3 bar). Bubbling is maintained for 30 minutes. During this time, turbidity will appear, indicating the formation of the cluster. After 30 minutes, the bubbling is stopped, and the solution/dispersion is left stirring for an additional 30 minutes. After this time, the mixture is collected into centrifuge tubes and centrifuged to remove the solvent and amine residues. The whitish supernatant is washed with ethanol and centrifuged again. This process is repeated four times. Once the supernatant is

clean, the residue is redispersed in the minimum amount of ethanol needed to prevent the cluster from settling at the bottom.

- Preparation of the $(CaCO_3)_n$ clusters: 200 mg of $CaCl_2 \cdot 2H_2O$ were added to 100 mL of ethanol and the mixture was sonicated to disperse the salt. After have being fully dissolved, the solution was placed into a 250 mL round flask with stirring (300 rpm) where 1.2 mL of TEA were added. After the addition of the amine, CO_2 started to flow from a reservoir to the flask through a capillary at 100 cm^3/min during 5-10 minutes. Upon bubbling, the solution becomes turbid, indicative of the $CaCO_3$ being formed, and becomes transparent again due to the amine, which is capping the carbonate chains and making them stable in the liquid phase. After the bubbling stops, the solution is kept in stirring for 10-30 more minutes. The stirring times after the exposure to CO_2 were dependent on the amount of time the solution required to shift back from white to transparent, which varied depending on the specific synthesis. The obtained solution is centrifuged at high speed (20.000 g), washed and rinsed 3 times with fresh ethanol.

2.2. Exploring the synthetic variables

Table S1. Summary of the effect of synthetic parameters on the formation of $MgCO_{3n}$ clusters.

Entry	Mg source	Base	Solvent	Result	Reference
**1**	$MgCl_2$	Triethylamine, Et_3N (TEA)	EtOH	Turbidity	Original synthetis $[MgCO_3]_n$-TEA
**2**		*N,N*-Diisopropylethylamine (DIPEA)		Turbidity	$[MgCO_3]_n$-DIPEA
**3**		N-Methylpyrrolidine (NMPy)		Turbidity	$[MgCO_3]_n$-NMPy
		1,4 Diazabicyclo[2.2.2]octane (DABCO)		Turbidity	$[MgCO_3]_n$-DABCO
**4**		Aniline		Clean solution	
**5**		Pyrrolidine		Clean solution	
**6**		KOAc		Clean solution	
**7**	MgI_2	Et_3N		Turbidity	$[MgCO_3]_n$-MgI_2
**8**	$Mg(OAc)_2$			Clean solution	
**9**	$MgCl_2$		H_2O	Clean solution	
**10**			H_2O/EtOH (1:1)	Clean solution	
**11**			MeOH	Clean solution	
**12**			IPA	Precipitate	$MgCl_2$-IPA

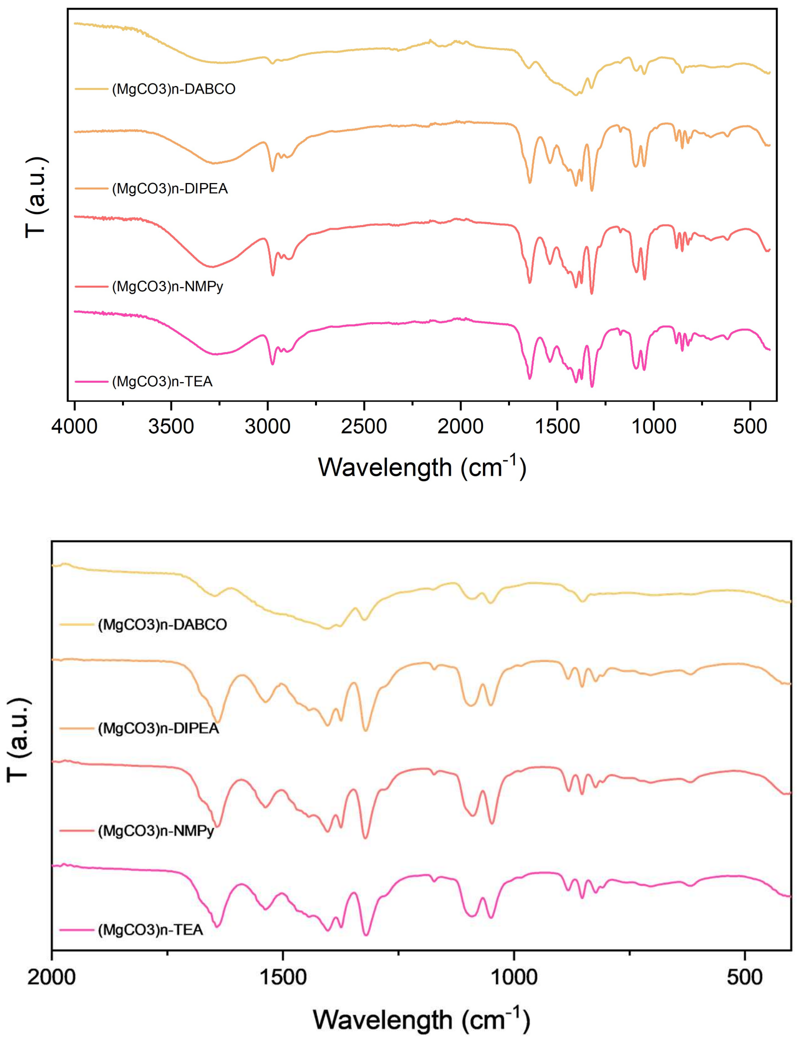

Figure S1. (top) Attenuated total reflection Fourier–transform infrared (FT–IR) general spectra of the $(MgCO_3)_n$ clusters. (bottom) Zoom from 2000 cm^{-1} to 450 cm^{-1} of spectra of the $(MgCO_3)_n$ clusters.

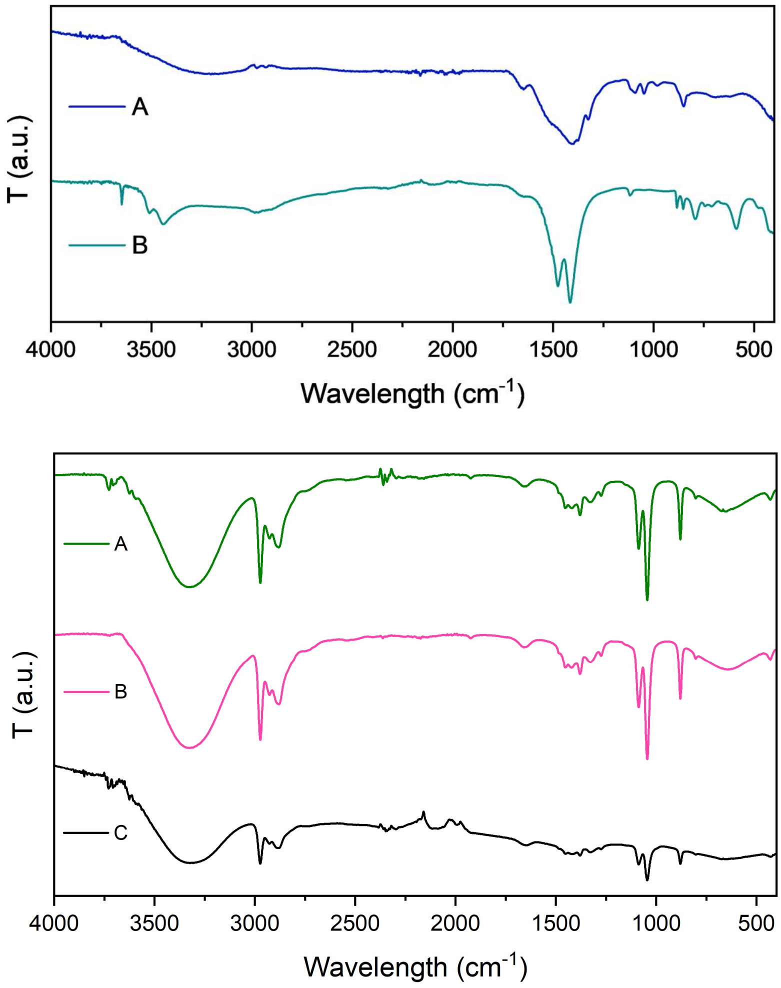

Figure S2. (top) Comparation of the attenuated total reflection Fourier–transform infrared (FT–IR) spectrum of the $(MgCO_3)_n$ clusters in dry gel form (blue line, A) with the bulk $MgCO_3$ (green line, B). (bottom) Fourier transform infrared (FT-IR) spectra of the redispersed $(MgCO_3)_n$ clusters in ethanol after heating at 110 ºC (green line, A), the $(MgCO_3)_n$ clusters in ethanol (pink line, B) compared with ethanol (black line, C). The spectra in the range of 400 cm^{-1} to 4000 cm^{-1} are nearly identical for both ethanol and the gel-like oligomers, confirming that ethanol is the primary component in the gel-like $(MgCO_3)_n$ oligomers.

Table S2. Results of elemental analysis for $(MgCO_3)_n$ clusters obtained under different synthetic conditions and for $(CaCO_3)_n$ as dry gels.

Entry	Compound	% C	% H	% N
1	$[MgCO_3]$n-TEA	14.49	3.87	0.01
2	$[MgCO_3]_n$-DIPEA	11.25	3.09	0.04
3	$[MgCO_3]_n$-NMPy	8.77	2.26	0.03
4	$[MgCO_3]_n$-DABCO	19.76	4.06	2.30
5	$[MgCO_3]_n$-MgI_2	13.51	3.08	0.00
6	$[CaCO_3]_n$	10.22	1.32	0.17

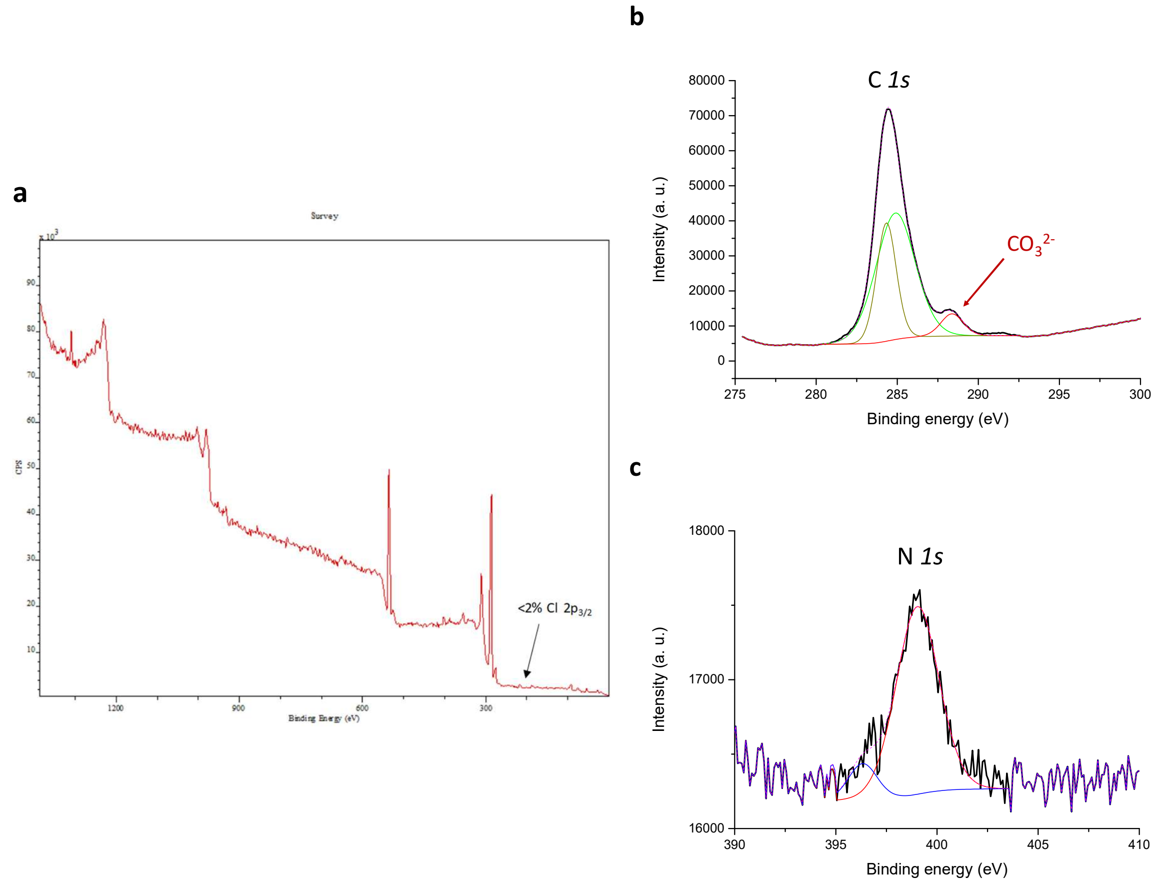

Figure S3. a) X-ray photoelectron spectrum (XPS) survey form, b) C 1s X-ray photoelectron spectrum (XPS) and c) N 1s X-ray photoelectron spectrum (XPS) for the $[MgCO_3]_{2\text{-}9}\cdot 2\text{-}6H_2O$ clusters in dry gel.

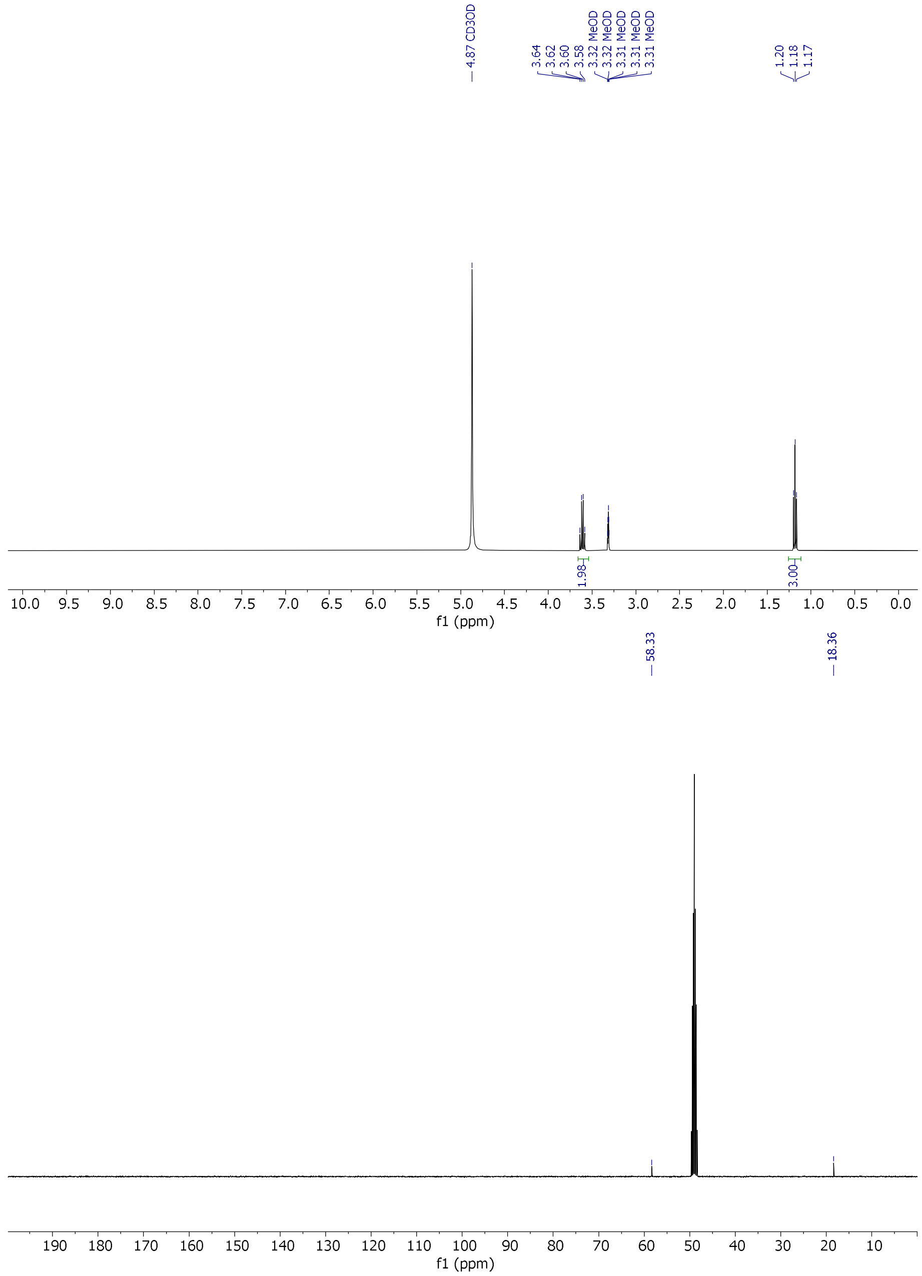

Figure S4. ^{1}H and ^{13}C nuclear magnetic resonance (NMR) of the $[MgCO_3]_{2\text{-}9}\cdot 2\text{-}6H_2O$ clusters after redissolving in CD_3OD.

Table S3. Dynamic Light Scattering (DLS) values of ethanol solution with $[MgCO_3]_n$ clusters obtained using different bases and the bulk $MgCO_3$.

Entry	Compound	Base	pKa	Average diameter (nm)
1	$[MgCO_3]_n$-TEA	TEA	10.7	1.3
2	$[MgCO_3]_n$-DIPEA	DIPEA	10.9	1.5
3	$[MgCO_3]_n$-NMPy	NMPy	10.3	13.5
4	$[MgCO_3]_n$-DABCO	DABCO	8.7	164.2
5	Bulk $MgCO_3$	---	---	1484.0

$MgCO_3$ bulk

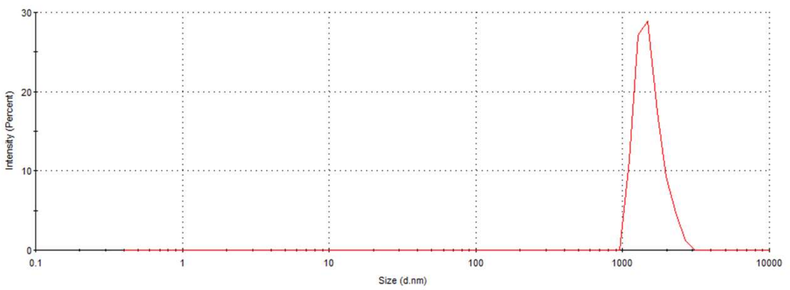

TEA

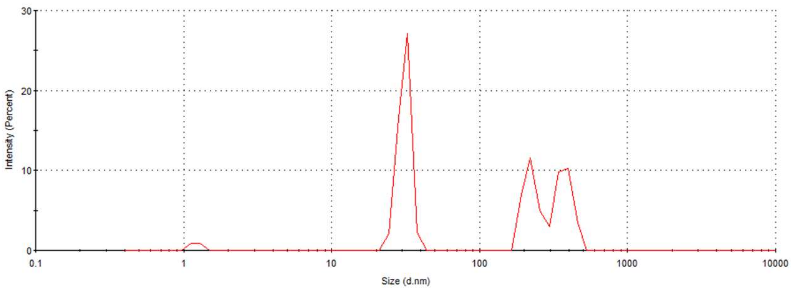

DIPEA

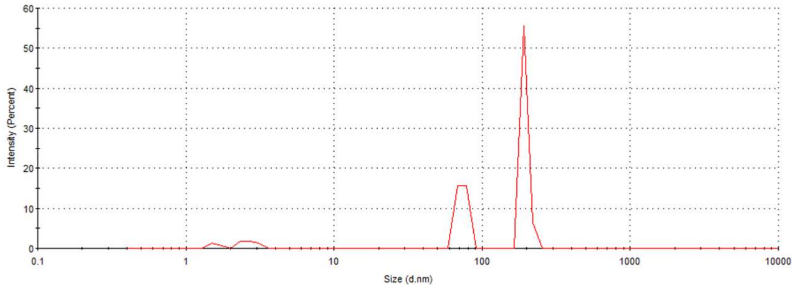

NMPy

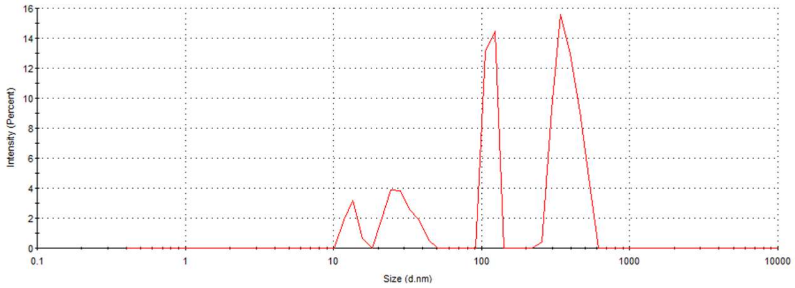

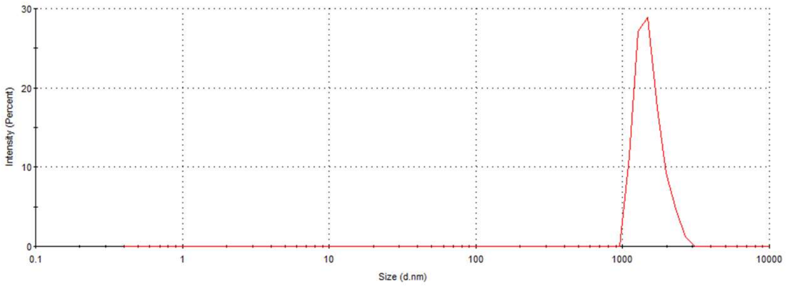

DABCO

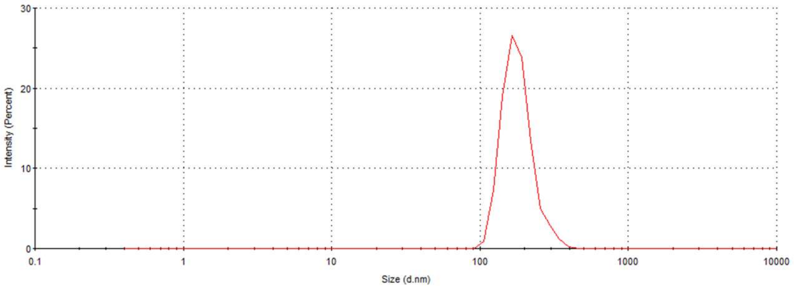

Figure S5. DLS size distribution and intensity $[MgCO_3]_n$ clusters obtained using different bases.

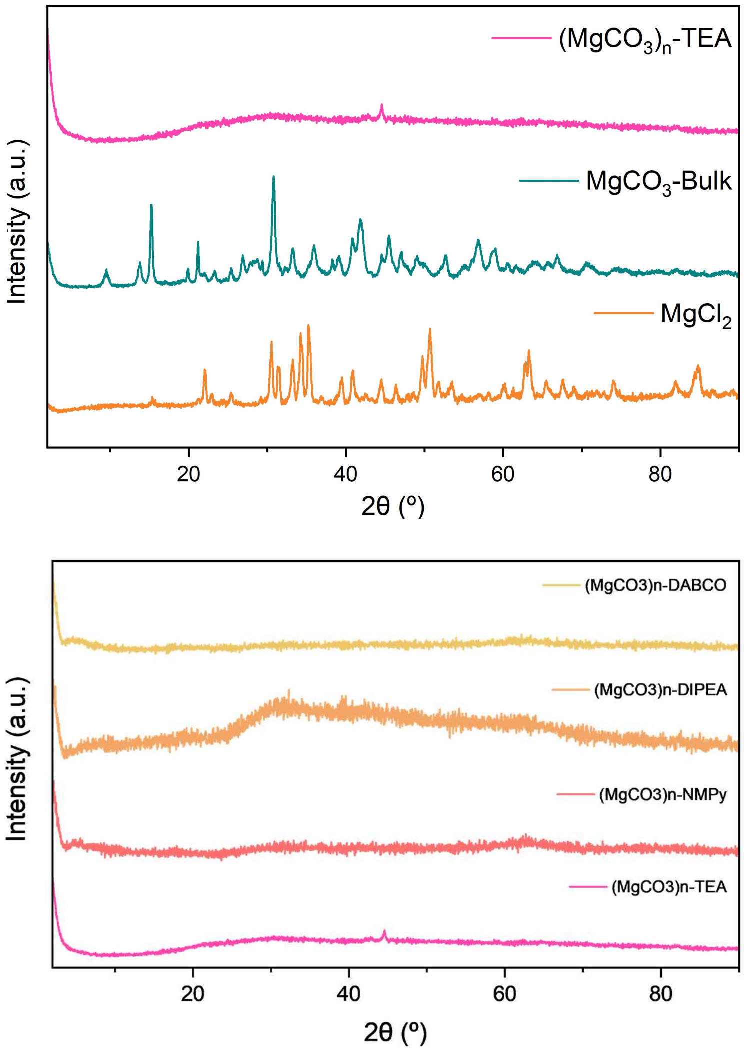

Figure S6. (top) X-ray diffractograms (PXRD) of (from bottom to top): the $[MgCO_3]_{2\text{-}9}\cdot 2\text{-}6H_2O$ clusters in dry gel form (pink line), the starting $MgCl_2$ solid (orange line) and the bulk $MgCO_3$ solid (blue line). (bottom) X-ray diffractograms (PXRD) of different terciary amino-based clusters.

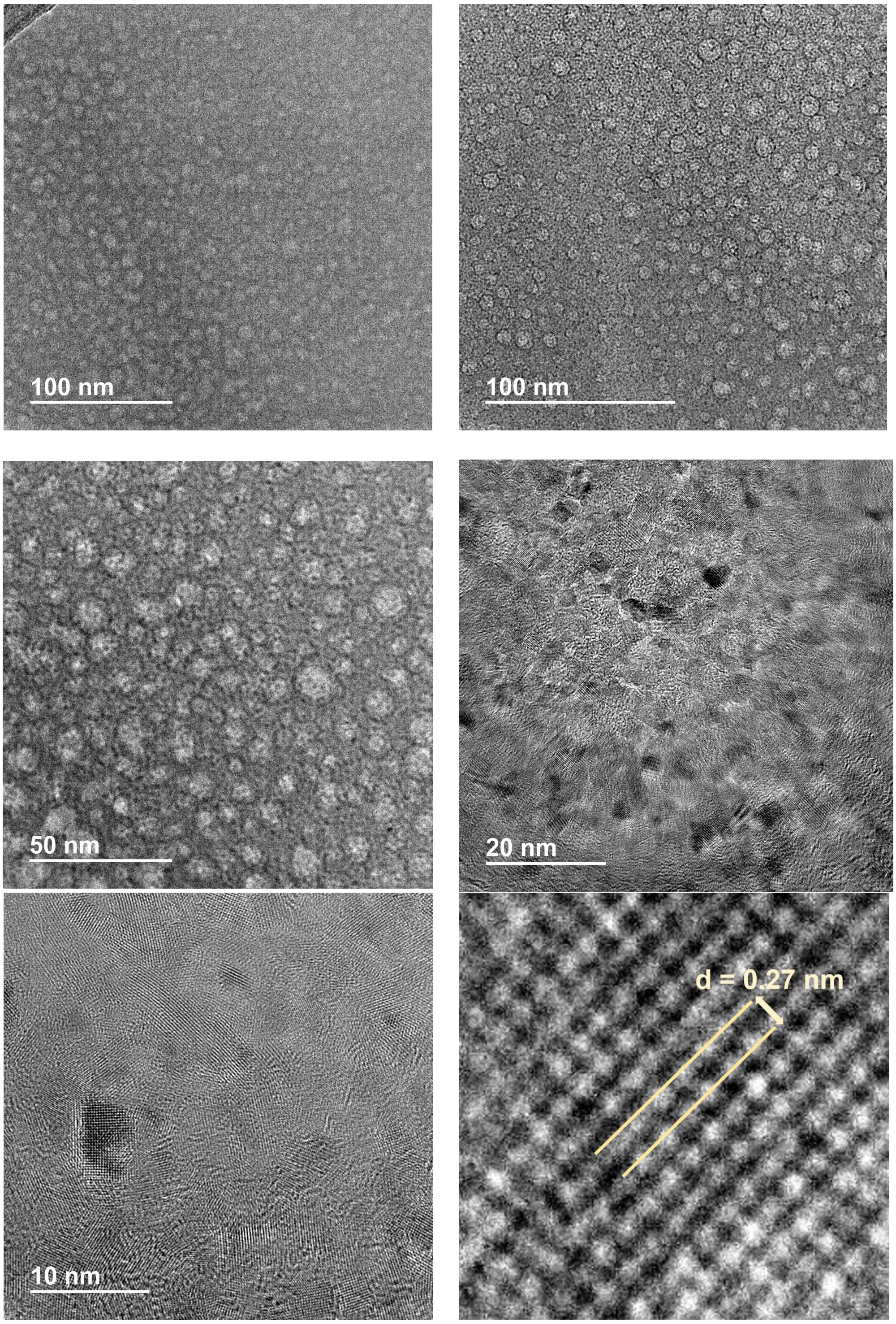

Figure S7. High resolution transmission electron microscopy (HR–TEM) images of the $[MgCO_3]_n$ clusters prepared with TEA, for different samples and at different magnifications.

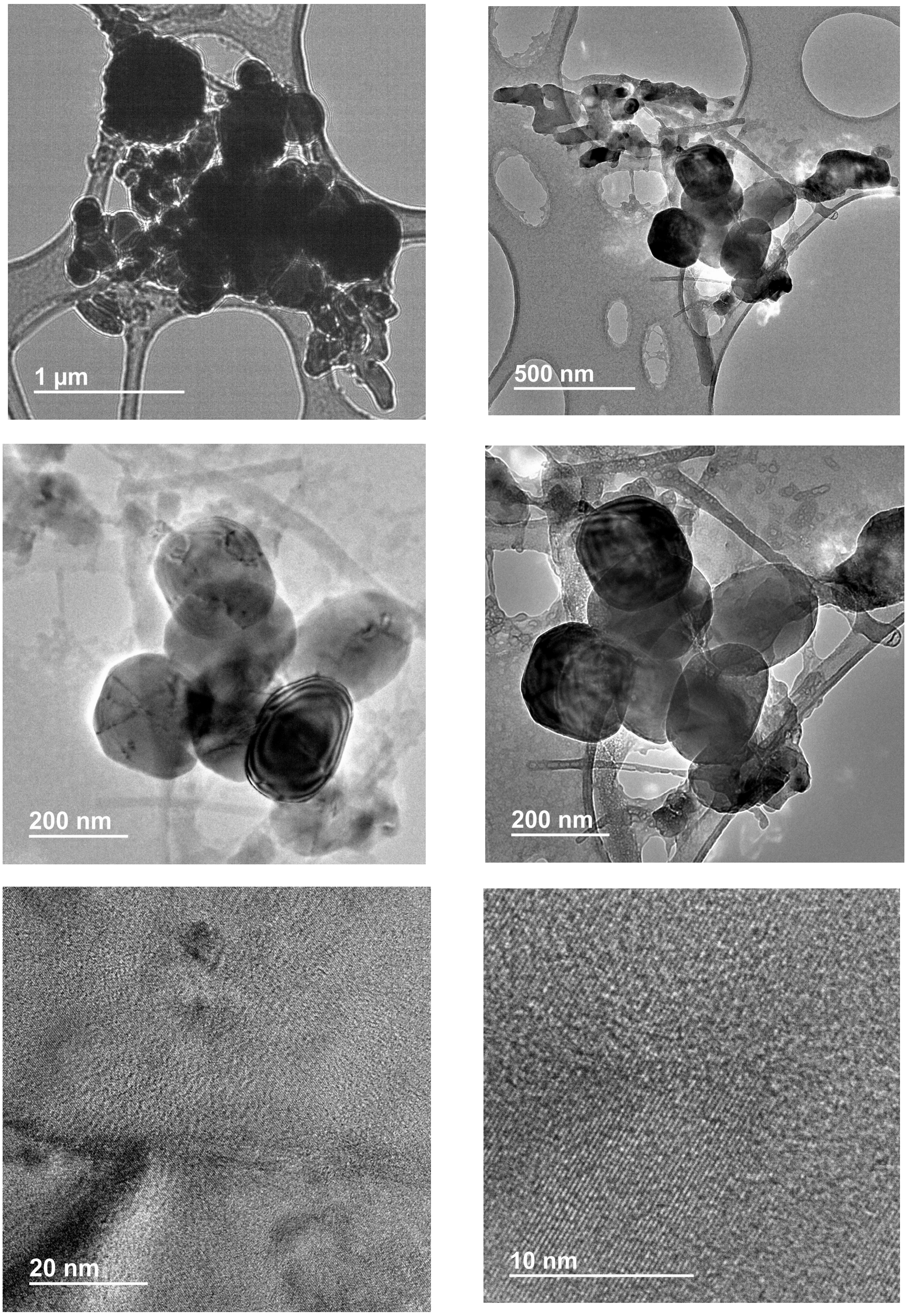

Figure S8. High resolution transmission electron microscopy (HR–TEM) images of the $[MgCO_3]_n$ clusters prepared with DABCO, for different samples and at different magnifications. The presence of organic material (DABCO) somehow blurres the images and hampers adqusition at high magnifications.

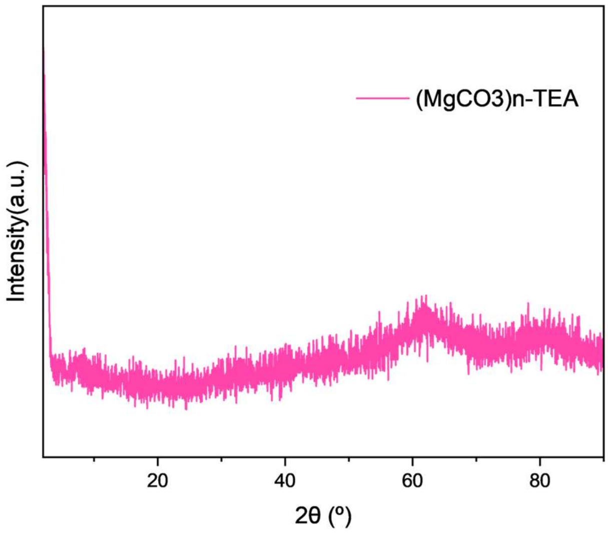

Figure S9. X-ray diffractogram (PXRD) of the $[MgCO_3]_{2\text{-}9}\cdot 2\text{-}6H_2O$ nanoclusters (prepared with TEA) after slow crystallization on the PXRD cell.

3. Computational modelling and comparison of the Ca^{+2} and Mg^{+2} carbonate clusters

3.1. Computational details

Initial Cartesian coordinates of the model systems were extracted from the Supporting Information of Dixon and co-workers,[S1] who previously carried out a systematic global minimum search for carbonate clusters of different sizes using a tree-growth hybrid genetic algorithm followed by DFT refinement. Specifically, the structure corresponding to the global minimum of the cluster composed of eight carbonate units was selected as the parent model.

All geometry optimizations and frequency calculations were performed using the ωB97X-D functional, a long-range corrected hybrid density functional including empirical dispersion, in combination with the def2-SVP basis set for all atoms, as implemented in Gaussian16.[S2] An ultrafine integration grid was employed throughout. Vibrational analyses confirmed the nature of the optimized structures as minimum on the potential energy surface, with no imaginary frequencies.

The exploration of possible interaction sites between the carbonate clusters and triethylamine (TEA) was carried out using the Glide docking code implemented in the Schrödinger suite.[S3] A cubic grid of 20 Å was defined around the cluster, enabling an unbiased and systematic exploration of all possible binding sites. Up to 20 poses were retained per docking run, using default force-field and sampling parameters, and all remaining settings were kept at their default values.

The lowest-energy docking poses were subsequently fully re-optimized at the same DFT level as the bare clusters. Reported complexation energies include corrections for basis set superposition error (BSSE), computed using the counterpoise method.[S4]

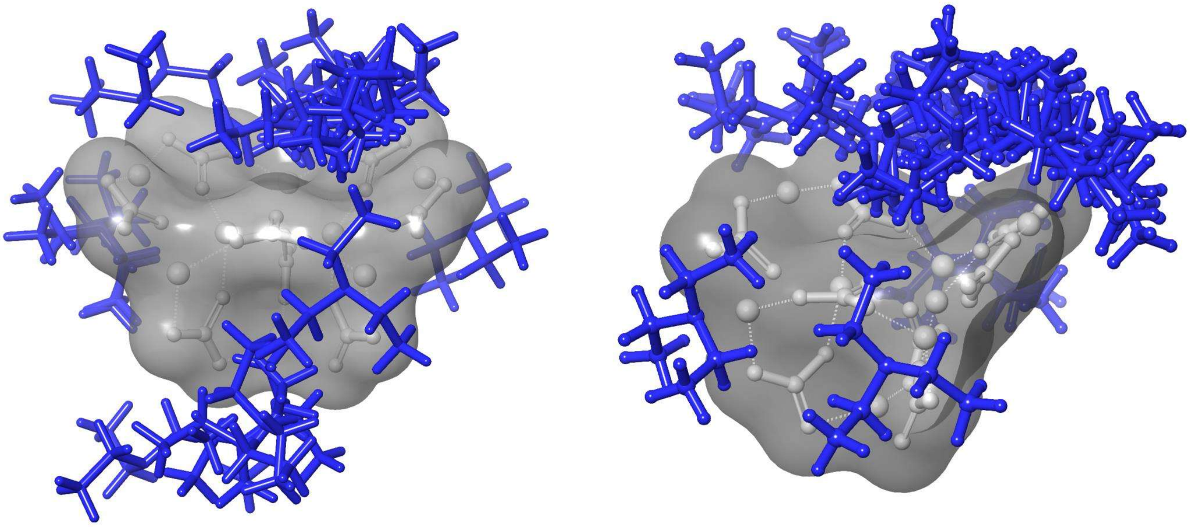

$[CaCO_3]_8$ – TEA $[MgCO_3]_8$ – TEA

Figure S10. Overlay of generated poses during docking simulation. Central clusters are displayed as grey surfaces and TEA molecules are showed as blue sticks.

3.2. Additional experiments

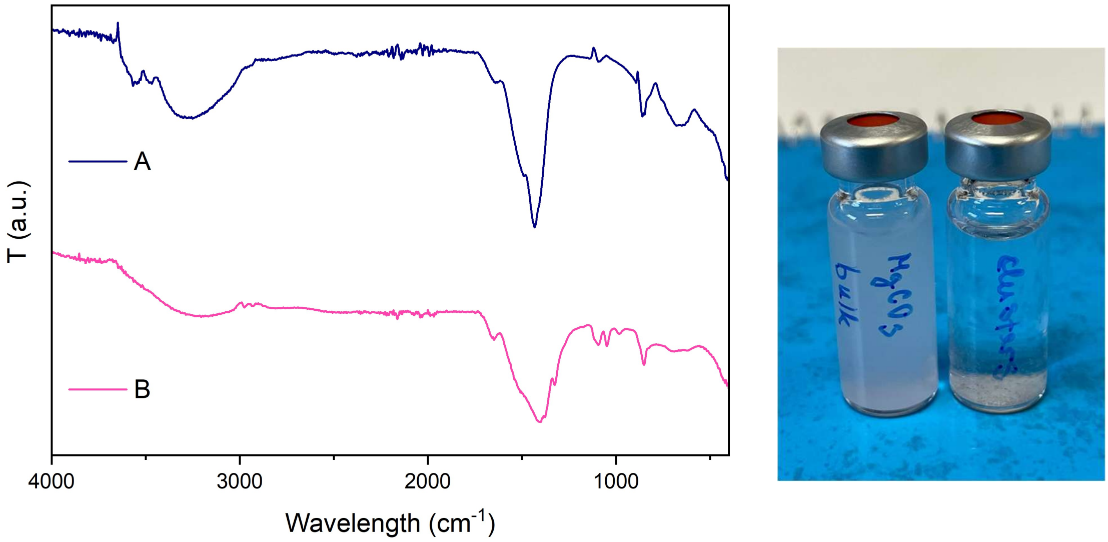

Figure S11. Fourier transform infrared (FT-IR) spectra of the $(MgCO_3)_n$ clusters in dry gel form before (B, pink line) and after (A, blue line) redispersion in water (left), and a photograph of the sedimented $MgCO_3$ materials in neat water after shacking and leaving to deposit at room temperature (right).

4. Catalytic reactions

- Typical reaction procedure [transesterification reaction of dimethyl carbonate (DMC, **1**) with ethanol]: DMC (84 μL, 1 mmol), the ethanolic solution of carbonate clusters (1.5 mol%) and additional EtOH until reaching 2 mL (34.3 mmol) were added into a 10 mL glass vial equipped with a magnetic stir. The vial was sealed and the resulting mixture was magnetically stirred over a temperature ranging from 40 to 110 °C on a heating plate for 22 h. The progress of the reaction was monitored by taking aliquots (25 μL) and analysing them by gas chromatography (GC) after dissolving in 1 mL of diethyl ether, filtering through a 25 μm polyamide filter, and adding n-dodecane (5.5 μL, 0.025 mmol) as an external standard. The conversion and yields were obtained from calibration curves. At the end of the reaction, the volatiles were removed under rotavapory vacuum suction, the remaining crude was redissolved in $CDCl_3$, the solids were filtered off, and the resulting product was weighted and analyzed by NMR.

- Reactions in MeOH: 5 mL of the ethanolic solution of $[MgCO_3]_n$ clusters were concentrated under rotavapory vaccum suction until a white gel was formed. The while gel was weighted to confirm that practically all the EtOH had been removed. Then, 5 mL of MeOH were added and the mixture was stirred or sonicated if necessary until the while gel was completely redissolved. This solution was employed to perform the reactions as indicated above.

- General procedure for scaling-up and kinetic measurements: DMC (420 μL, 5 mmol), the ethanolic solution of carbonate clusters (corresponding volume of 1.5 or 0.6 mmol%) and additional EtOH upon reaching 10 mL (172 mmol) were added into a 20 mL double-reinforced glass vial equipped with a magnetic stir. The vial was sealed with a septum and the resulting mixture was magnetically stirred at 110 °C on a heating plate for 22 h. The progress of the reaction was monitored by taking aliquots (50 μL) and analysing them by gas chromatography (GC) after dissolving in 1 mL of diethyl ether, filtering through a 25 μm polyamide filter, and adding n-dodecane (11 μL, 0.05 mmol) as an external standard.

4.1. Kinetics results

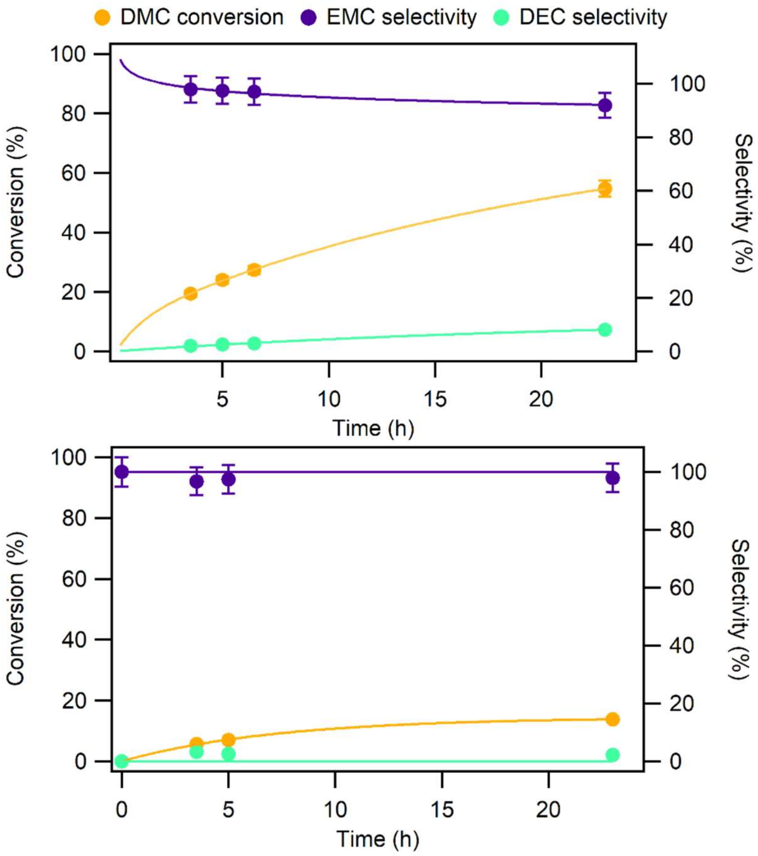

Figure S12. Kinetics profiles of $[MgCO_3]_n$ (top) and $[CaCO_3]_n$ clusters (bottom) at 80 ºC using 1.5 mol% of catalyst (see Table 1, entries 6 and 7, respectively). GC results. Error bars account for a 5% uncertainty.

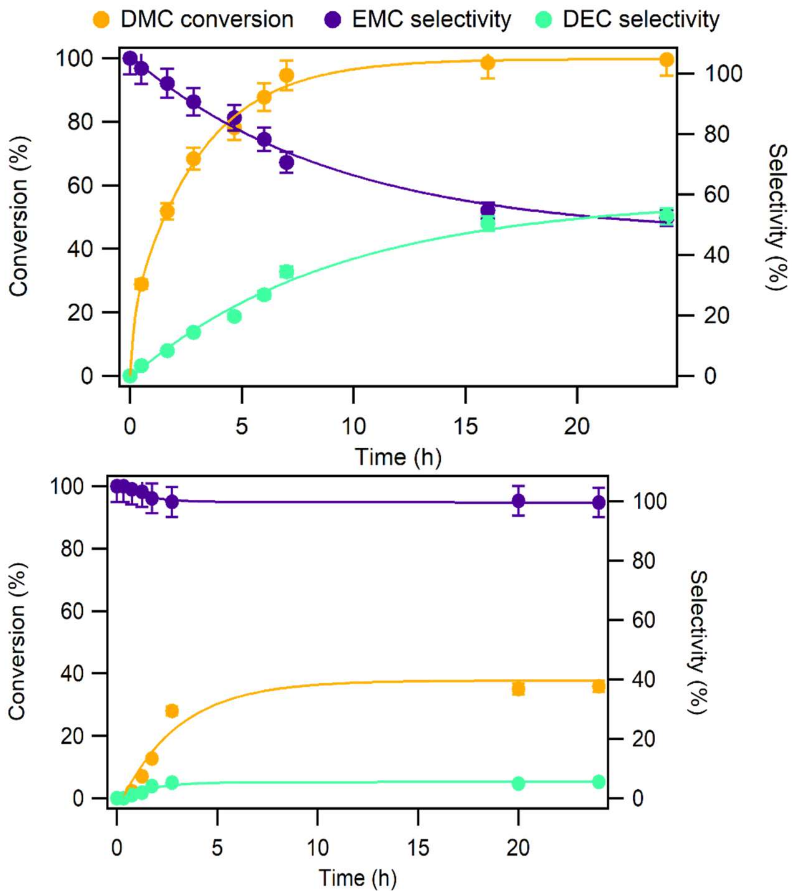

Figure S13. Kinetics profiles of $[MgCO_3]_n$ clusters (top) and bulk $MgCO_3$ (bottom) at 110 ºC using 1.5 mol% of catalyst (see Table 1, entries 9 and 10, respectively). GC results. Error bars account for a 5% uncertainty.

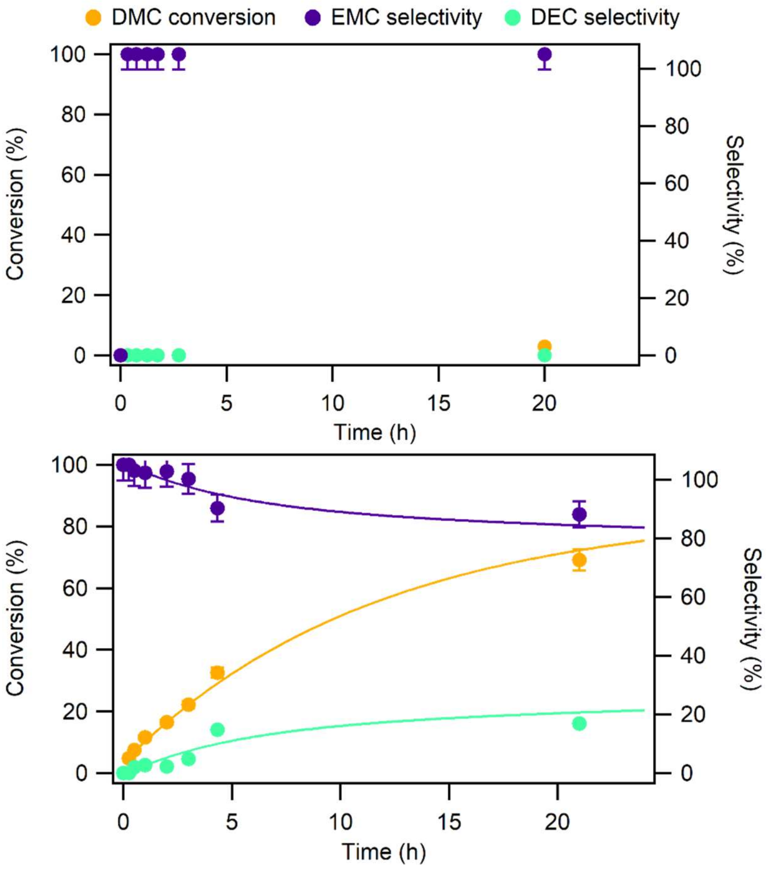

Figure S14. Kinetics profiles of bulk $CaCO_3$ (top) $[CaCO_3]_n$ clusters (bottom) at 110 ºC using 1.5 mol% of catalyst (see Table 1 entries 11 and 12, respectively). GC results. Error bars account for a 5% uncertainty.

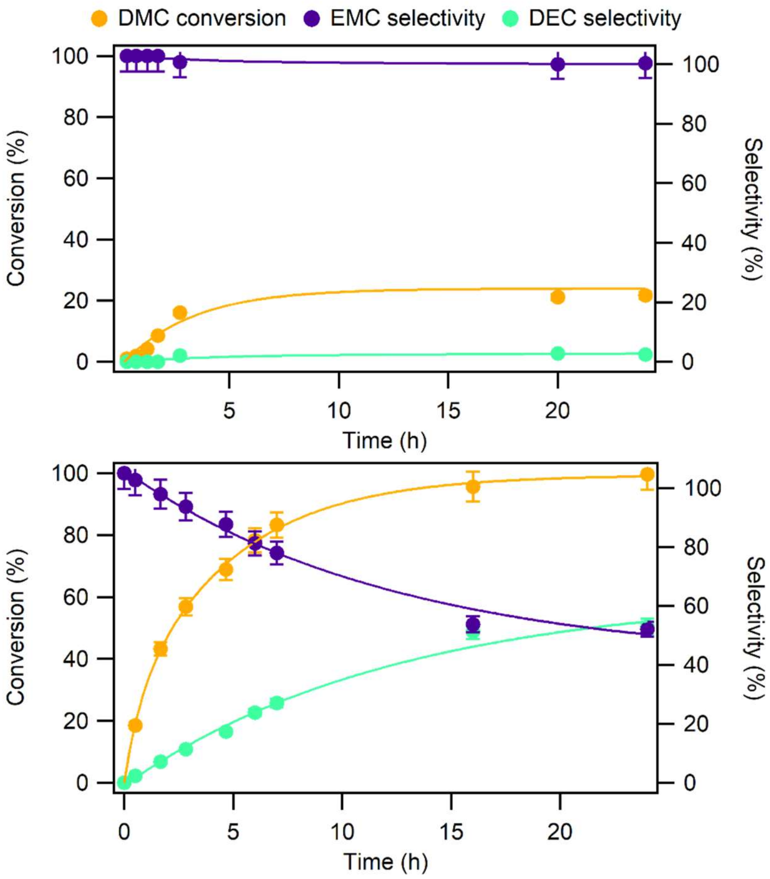

Figure S15. Kinetics profiles of bulk $MgCO_3$ (top) and $[MgCO_3]_n$ clusters (bottom) at 110 ºC using 0.6 mol% of catalyst (see Table 1, entries 14 and 13, respectively). GC results. Error bars account for a 5% uncertainty.

Table S4. List of catalytic parameters for the different materials under the optimized reactions conditions at 110 ºC. [a] TOF_0 refers to the turnovers effected by carbonate clusters considering, in average, 5 units of carbonate per cluster in the case of magnesium and 8 units for calcium cluster.

Entry	Material	Quantity (mg)	Mol%	Initial rate (%·h^{-1})	TOF_0 (h^{-1})[a]
1	$(MgCO_3)_5 \cdot 5H_2O$	0.57	0.6	25.2	210
2	$MgCO_3$ bulk	0.57	0.6	5.1	9
3	$(MgCO_3)_5 \cdot 5H_2O$	1.42	1.5	30.9	103
4	$MgCO_3$ bulk	1.42	1.5	9.7	6
5	$(CaCO_3)_8$	1.42	1.5	9.1	49
6	$CaCO_3$ bulk	1.42	1.5	< 1	<5

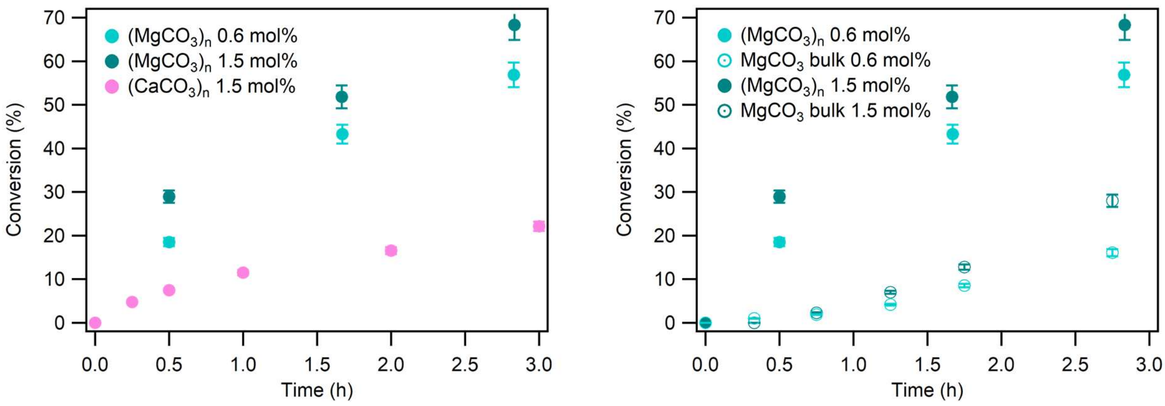

Figure S16. (right) Comparison of the kinetics profiles until 3 h reation time for the $[MgCO_3]_n$ and $[CaCO_3]_n$ clusters at 110 ºC, with different quantities of catalyst. (left) Comparison of the kinetics profiles until 3 h reation time for bulk $MgCO_3$ and the $[MgCO_3]_n$ clusters at 110 ºC, with different quantities of catalyst. GC results. Error bars account for a 5% uncertainty.

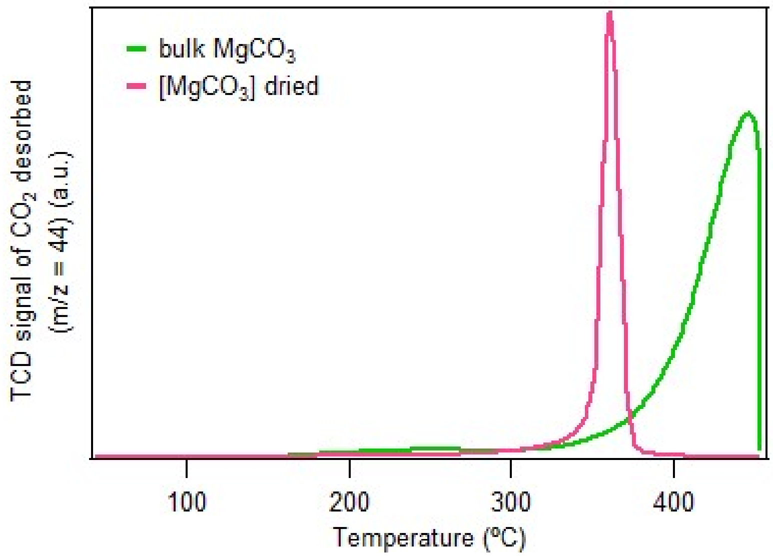

Figure S17. Carbon dioxide temperature-programmed desorption CO2-TPD of $[MgCO_3]n$ dried clusters and bulk $MgCO_3$.

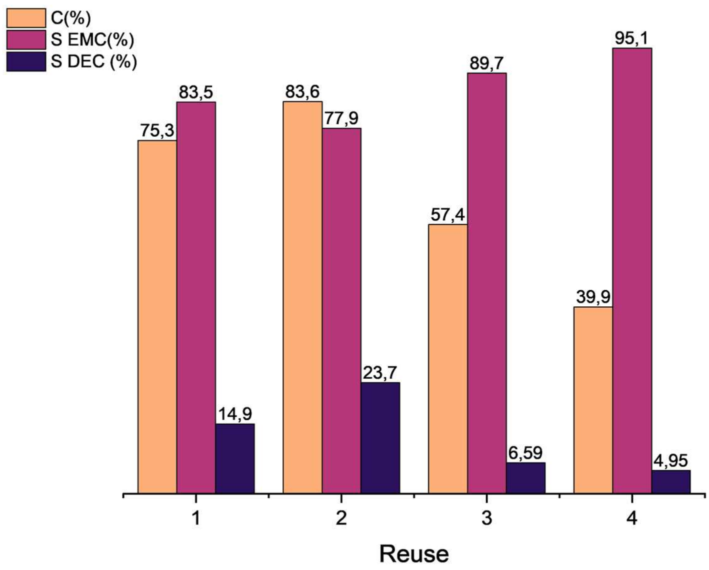

Figure S18. Reuses for the transesterification reaction of DMC 1 (1 mmol) with ethanol (0.5M) catalyzed by $[MgCO_3]_n$ at 110 ºC after 18 h of reaction. GC results. The final conversion is not complete under selected reaction conditions, which is more convenient for a reusability study.

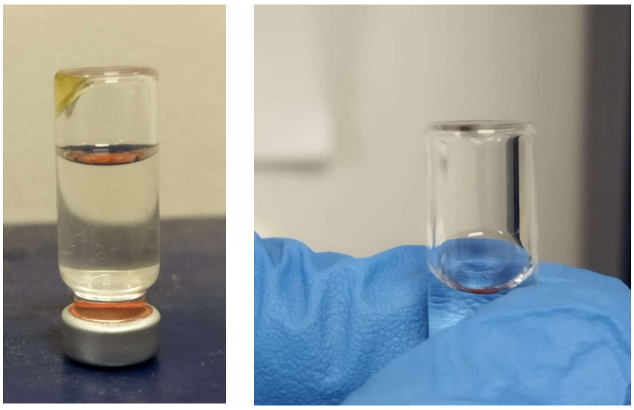

Figure S19. Progressive catalyst mass loss observed over successive reuse cycles due to the challenges associated with handling the material as an ethanolic gel. (Left) Vial after the first use with the clusters following centrifugation; (Right) vial after the fourth use, showing a clear decrease in catalyst mass after centrifugation

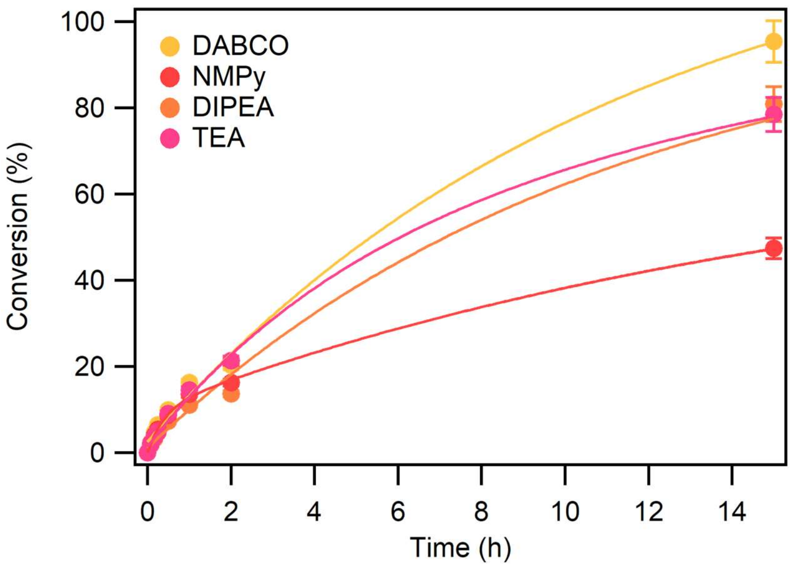

Figure S20. Kinetic profile for the transesterification reaction of DMC 1 with ethanol catalyzed by $[MgCO_3]_n$ clusters prepared by different bases (0.45 mol%) at 110 ºC. GC results. Error bars account for a 5% uncertainty.

4.2. Reactive experiments

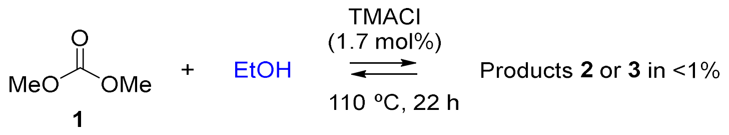

Scheme S1. Catalytic result with trimethylammonium chloride (TMACl) as a catalyst for the transesterification reaction of DMC **1** with ethanol (0.5M) at 110 ºC after 22 h reaction time. GC result.

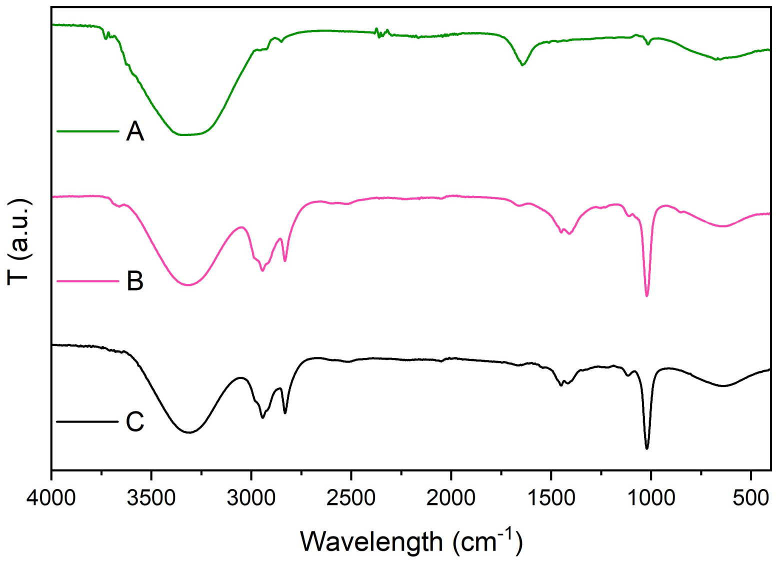

Figure S21. Fourier transform infrared (FT-IR) spectra of the redispersed $(MgCO_3)_n$ clusters in methanol after heating at 110 ºC O.N.(A), the redispersed $(MgCO_3)_n$ clusters in methanol (B), and methanol (C).

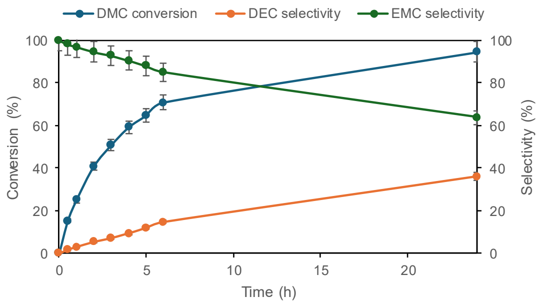

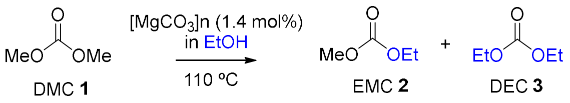

	Conversion of **1** (%)	Yields of **2** / **3** (%)
Original	90	29 / 61
Scale-up	95	31 / 64

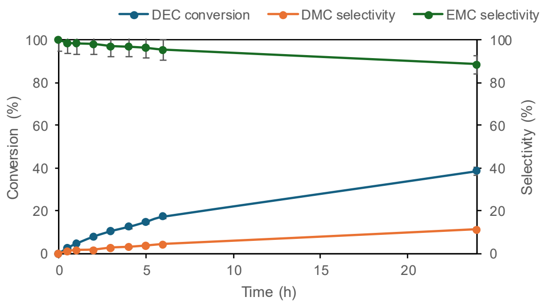

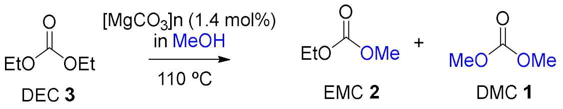

	Conversion of **3** (%)	Yields of **2** / **1** (%)
Original	45	7 / 38
Scale-up	39	4 / 35

Figure S22. Kinetic results for the transesterification reaction of the carbonate DMC **1** with EtOH (top) or DEC **3** with MeOH (bottom), at 1.25 mmol scale (0.5M), catalyzed by the $[MgCO_3]_n$ clusters (1.5 mol%) at 110 ºC. GC results. Error bars account for a 5% uncertainty.

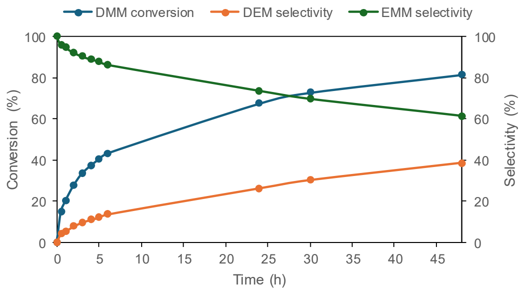

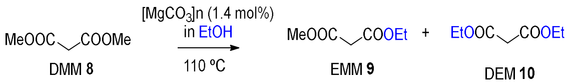

	Conversion of **8** (%)	Yields of **9** / **10** (%)
original	98	26 / 72
scale up	83	50 / 33

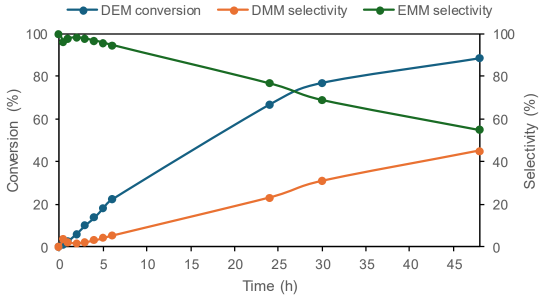

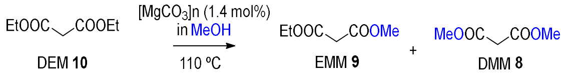

	Conversion of **10** (%)	Yields of **9** / **8** (%)
original	99	17 / 82
scale up	88	48 / 40

Figure S23. Kinetic results for the transesterification reaction of the malonate DMM **8** with EtOH (top) or DEM **10** with MeOH (bottom), at 1.25 mmol scale (0.5M), catalyzed by the $[MgCO_3]_n$ clusters (1.5 mol%) at 110 ºC. GC results. Error bars account for a 5% uncertainty.

4.3. NMR characterization

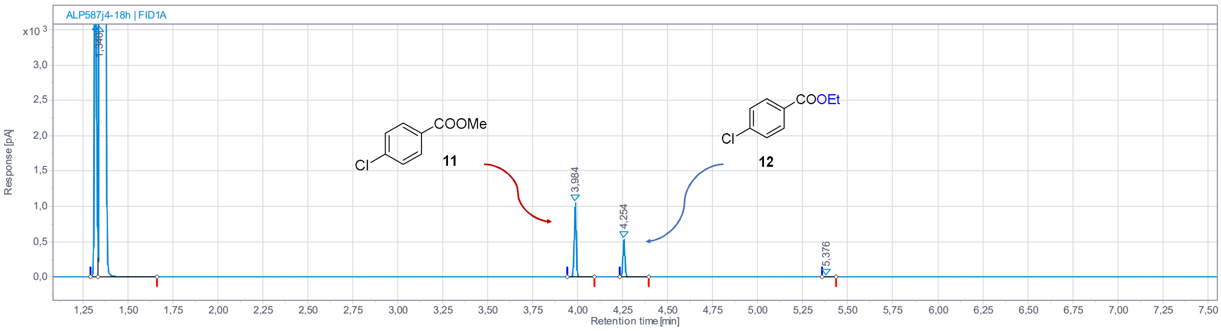

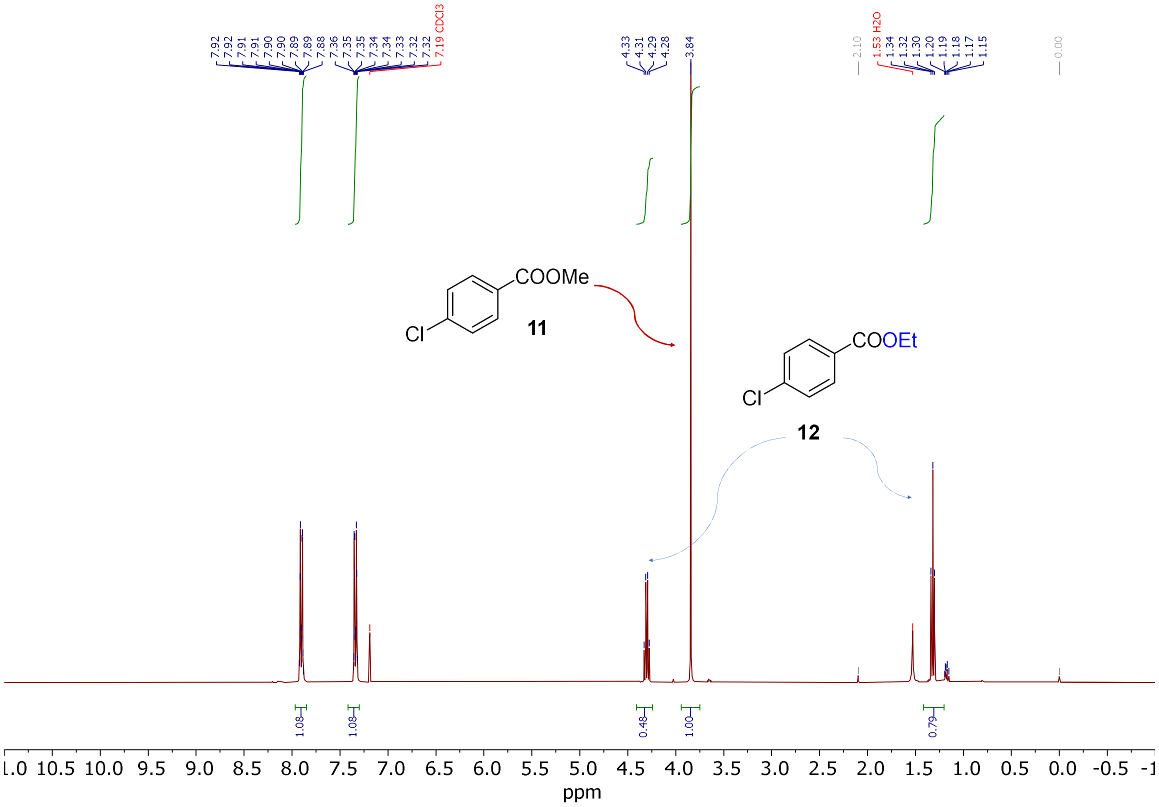

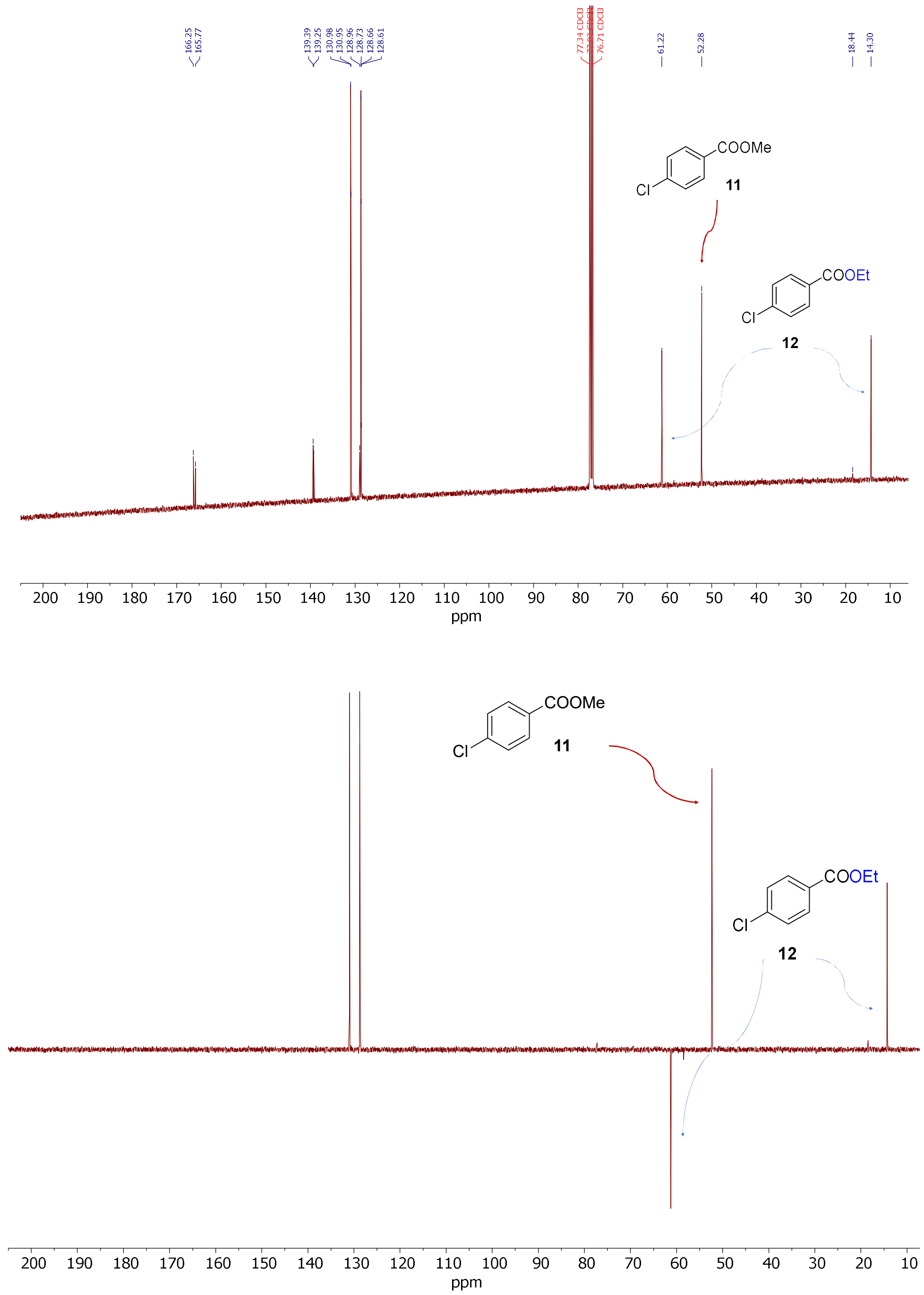

Figure S24. Gas chromatogram (top), ^{1}H (middle), ^{13}C (midlle) and distortionless enhancement by polarization transfer (DEPT, bottom) nuclear magnetic resonance (NMR) spectra of the product mixture corresponding to the transesterification reaction of 4-chloro methyl benzoate **11** (0.25 mmol) with ethanol (0.5M) catalyzed by $[MgCO_3]_n$ clusters (1.5 mol%) at 110 ºC for 22 h, after removing the volatiles, re-dissolving in $CDCl_3$ and filtering off the $[MgCO_3]_n$ clusters.

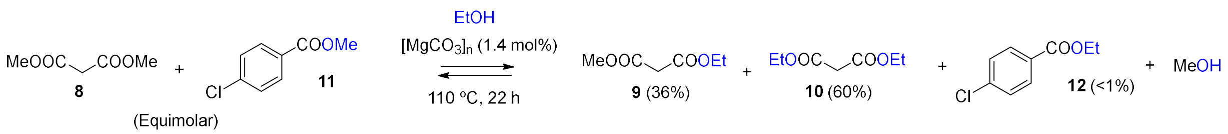

Figure S25. Results for the transesterification reaction of an equimolecular mixture of dimethyl malonate **8** and 4-chloro methyl benzoate **11** (0.25 mmol in total) with ethanol (0.5M) catalyzed by $[MgCO_3]_n$ clusters (1.5 mol%) at 110 ºC for 22 h. GC yields.

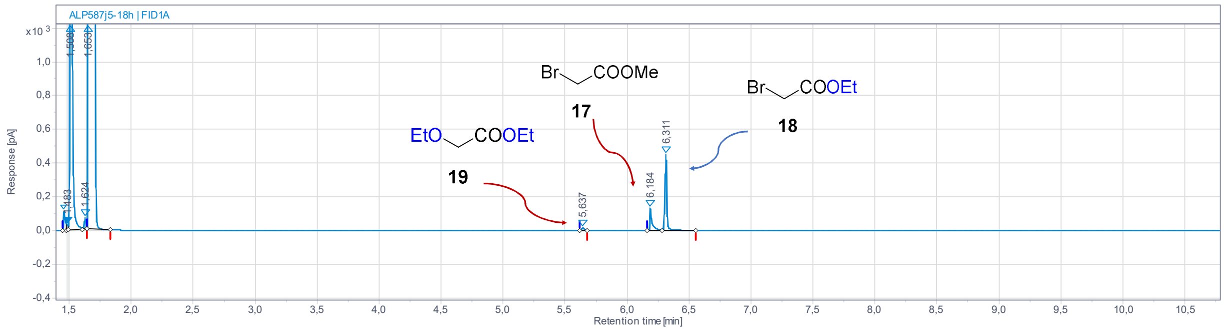
ALP587j5-18h | FID1A
Response [pA]
Retention time [min]
Br COOMe
17
Br COOEt
18
EtO COOEt
19

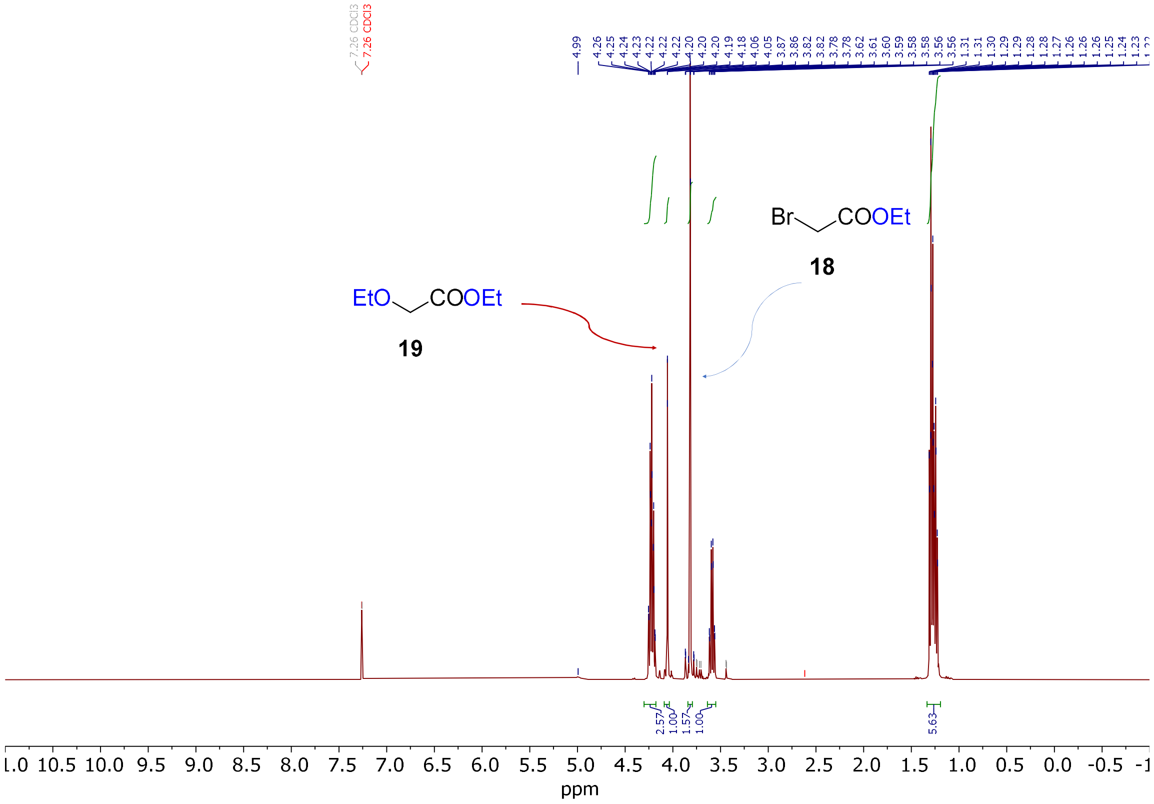
EtO COOEt
19
Br COOEt
18
ppm

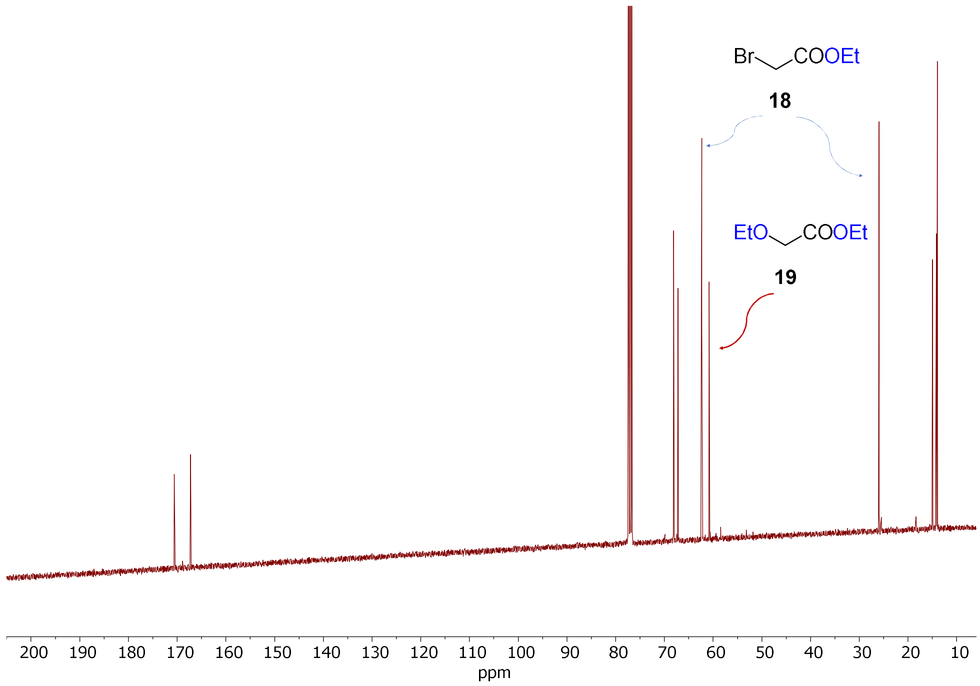

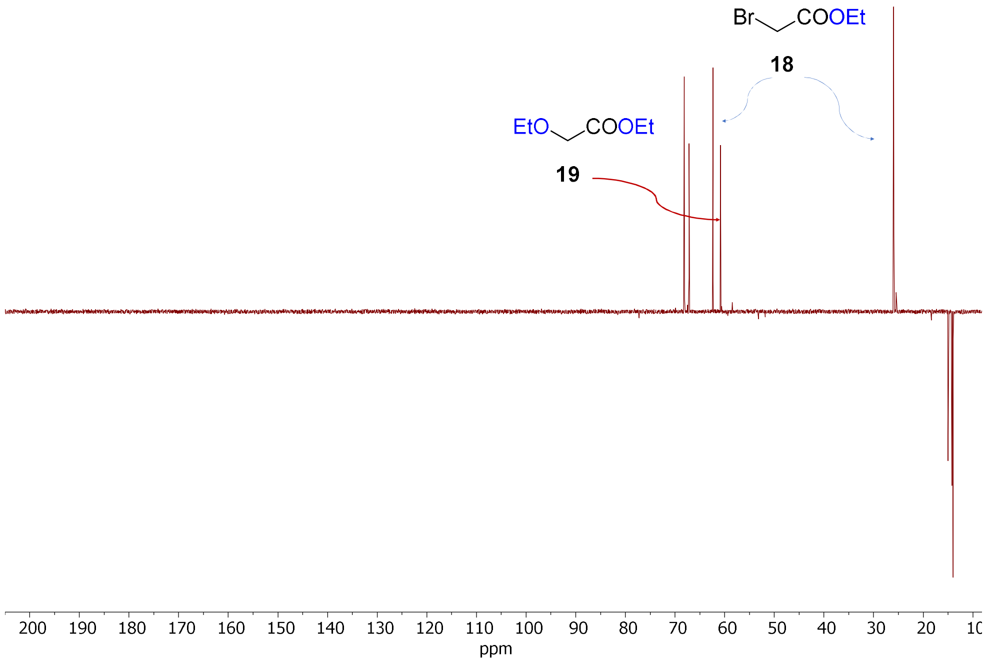

Figure S26. Gas chromatogram (top, 0.6 mol% catalyst), and ^{1}H (middle), ^{13}C (midlle) and distortionless enhancement by polarization transfer (DEPT, bottom) nuclear magnetic resonance (NMR) spectra (1.5 mol% catalyst) of the product mixture corresponding to the transesterification reaction of bromo methyl acetate **17** (0.25 mmol) with ethanol (0.5M) catalyzed by $[MgCO_3]_n$ clusters (1.5 mol%) at 110 ºC for 22 h, after removing the volatiles, re-dissolving in $CDCl_3$ and filtering off the $[MgCO_3]_n$ clusters.

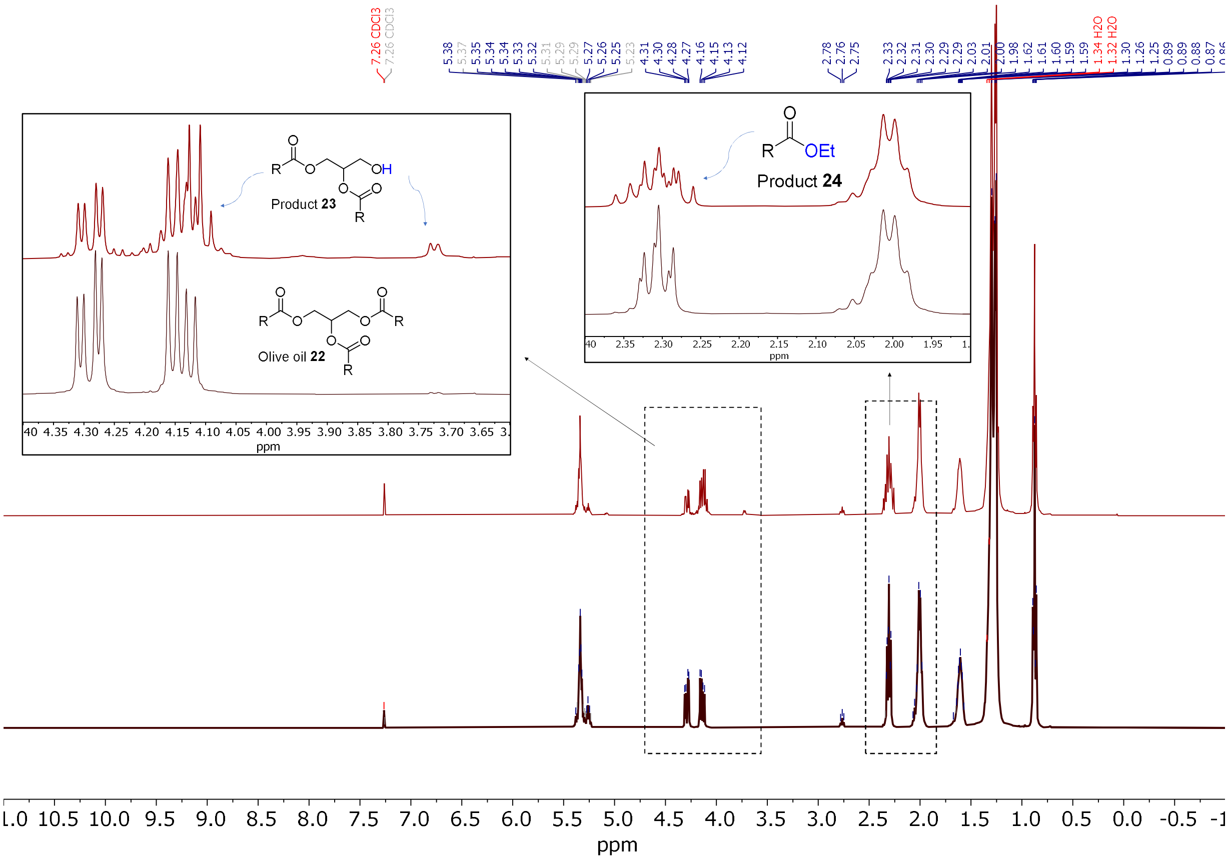

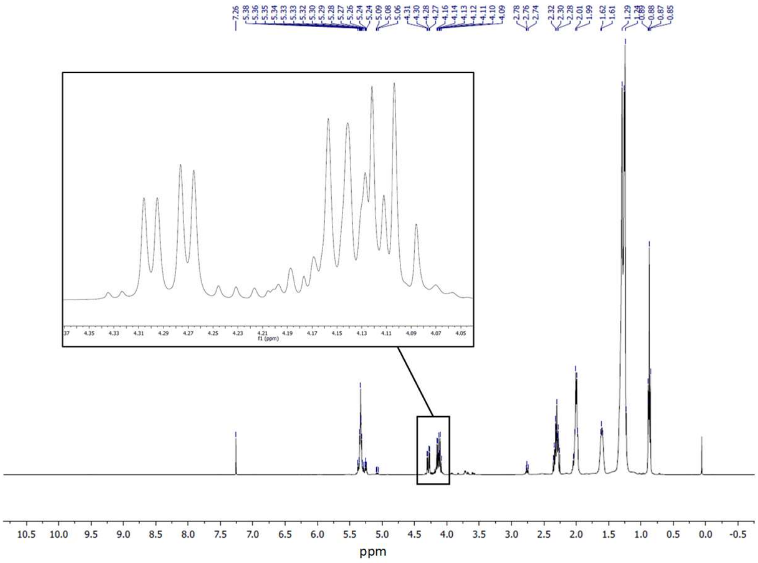

Figure S27. ^{1}H nuclear magnetic resonance (NMR) spectra of the product mixture corresponding to the ethanolysis reaction of a commercial sample of olive oil **22** (0.25 mmol) with ethanol (0.5M) catalyzed by $[MgCO_3]_n$ clusters (5 mol%) at 110 ºC for 22 h (top) and at 170 ºC for 46 h (bottom), after removing the volatiles, re-dissolving in $CDCl_3$ and filtering off the $[MgCO_3]_n$ clusters (top, red line), compared with starting material (bottom, brown line). The insets show the diagnostic areas.

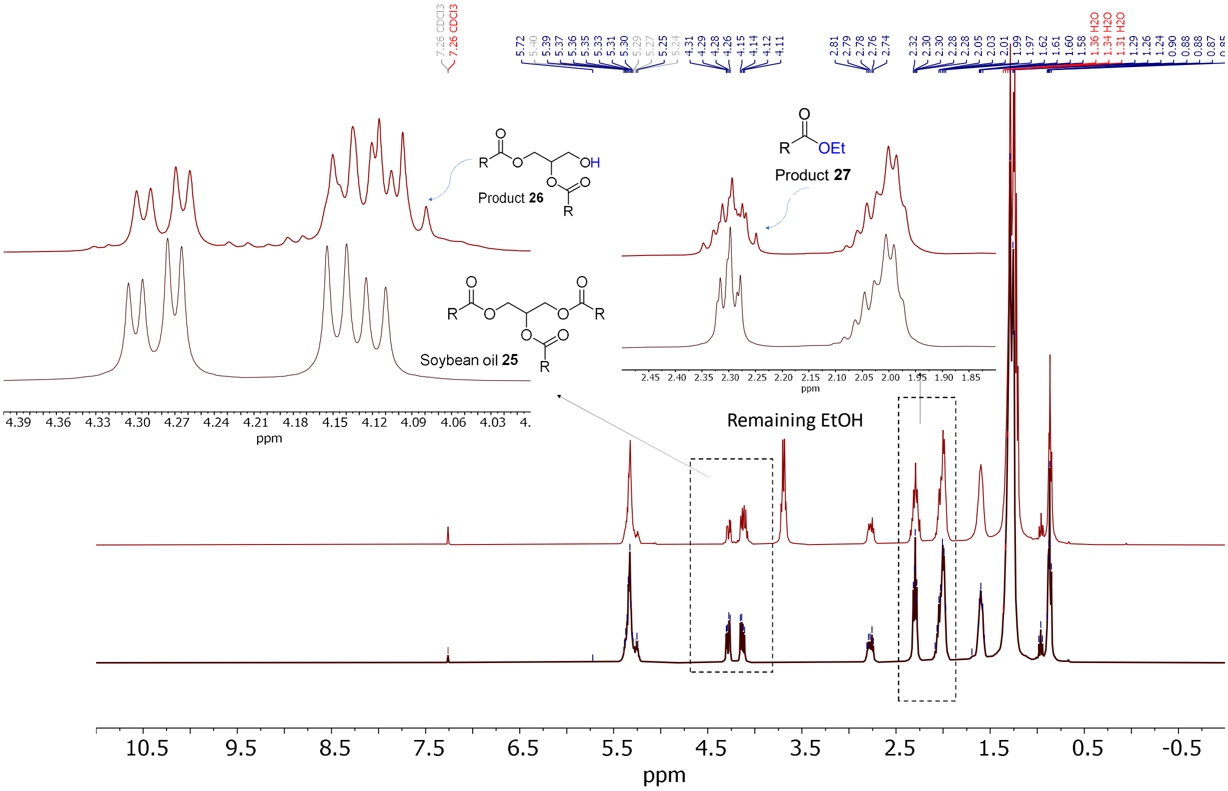

Figure S28. ^{1}H nuclear magnetic resonance (NMR) spectra of the product mixture corresponding to the ethanolysis reaction of a commercial sample of soybean oil **25** (0.25 mmol) with ethanol (0.5M) catalyzed by $[MgCO_3]_n$ clusters (5 mol%) at 110 ºC for 22 h, after removing the volatiles, re-dissolving in $CDCl_3$ and filtering off the $[MgCO_3]_n$ clusters (top), compared with starting material (bottom). The insets show the diagnostic areas.

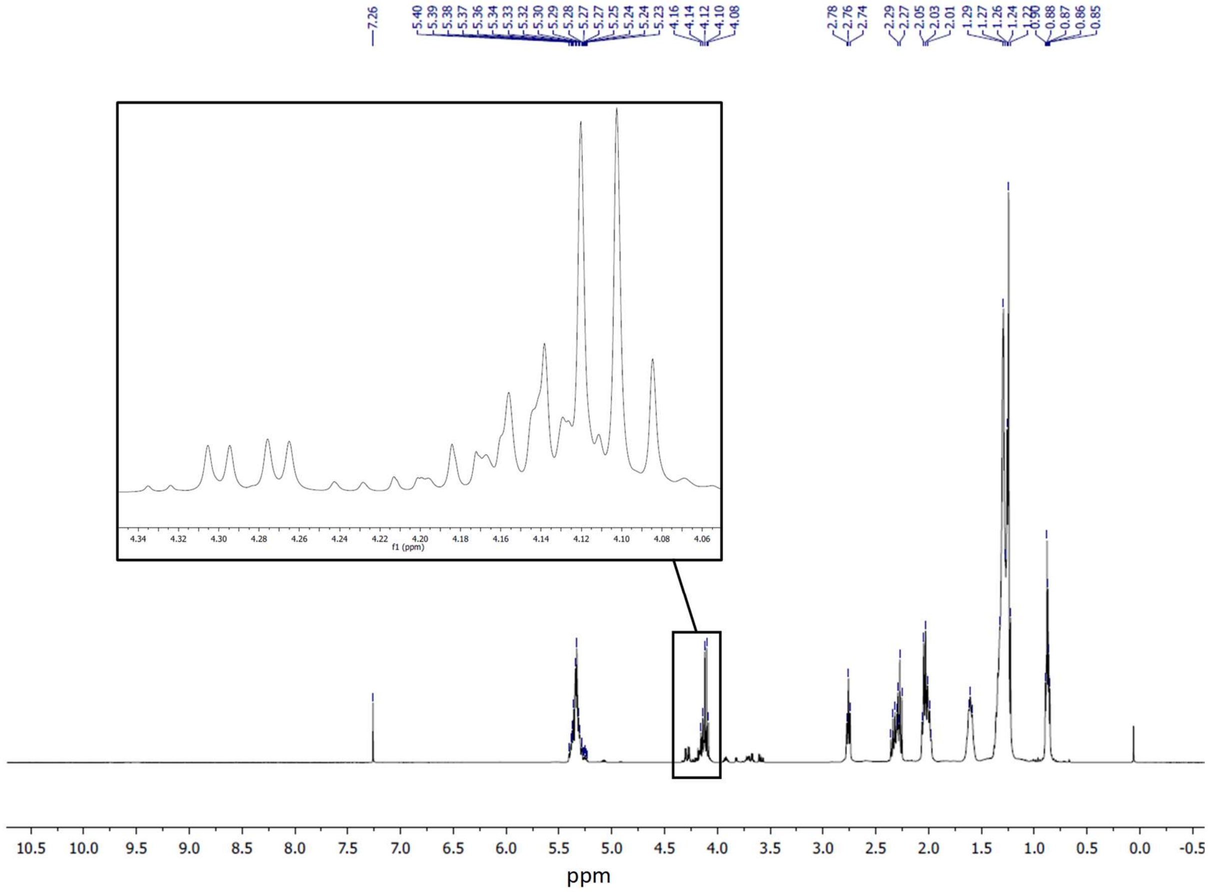

Figure S29. ^{1}H nuclear magnetic resonance (NMR) spectra of the product mixture corresponding to the ethanolysis reaction of a commercial sample of corn oil **28** (0.25 mmol) with ethanol (0.5M) catalyzed by $[MgCO_3]_n$ clusters (5 mol%) at 170 ºC for 46 h, after removing the volatiles, re-dissolving in $CDCl_3$ and filtering off the $[MgCO_3]_n$ clusters. The inset shows the diagnostic area.

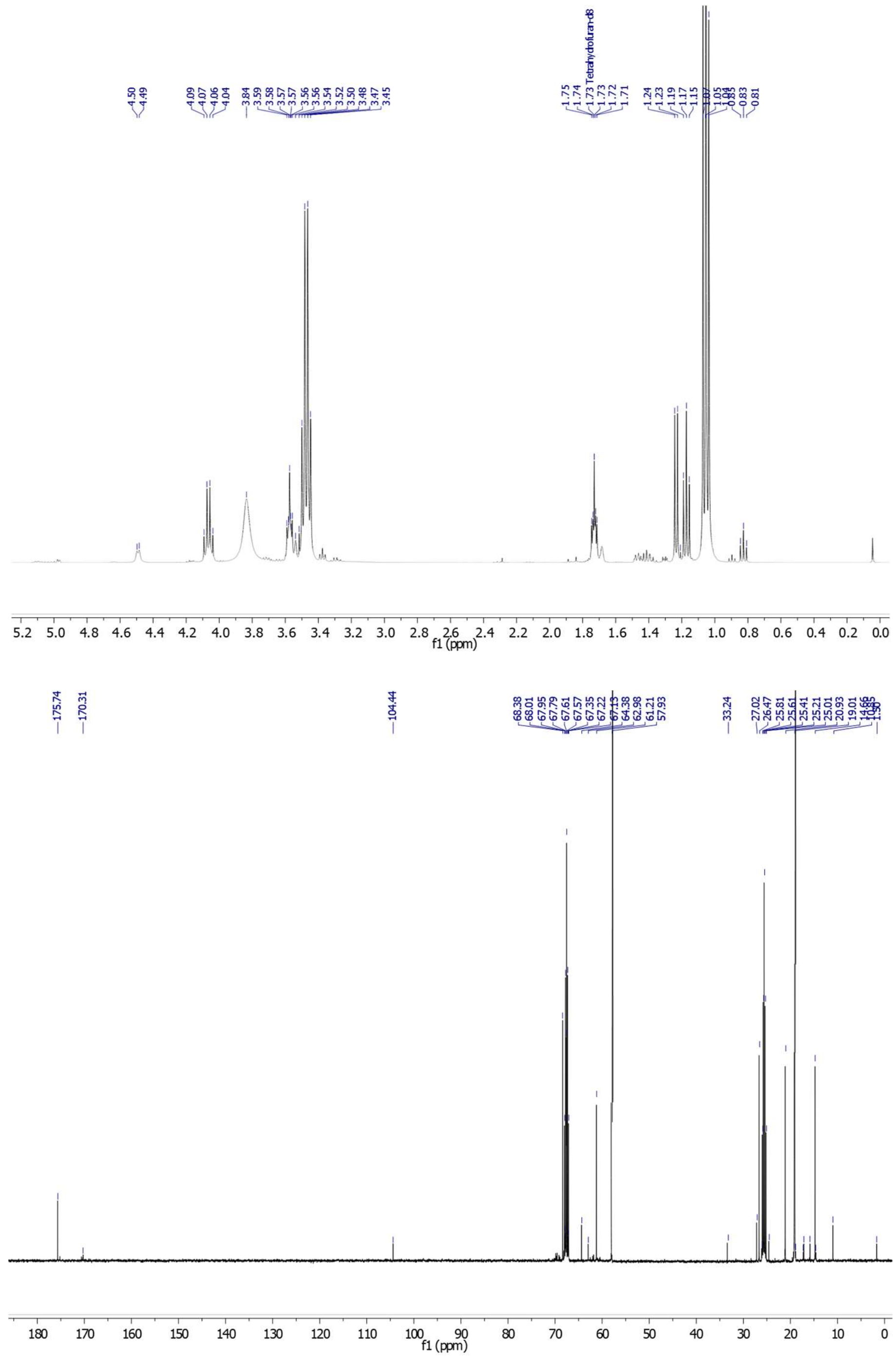

Figure S30. ^{1}H (top) and ^{13}C (bottom) nuclear magnetic resonance (NMR) spectra of the product mixture corresponding to the ethanolysis reaction of a commercial sample of polylactic acid **31** (0.25 mmol) with ethanol (0.5M) catalyzed by $[MgCO_3]_n$ clusters (5 mol%) at 170 ºC for 46 h, after removing the volatiles, re-dissolving in THF-d^8 and filtering off the $[MgCO_3]_n$ clusters. The formation of oligomers can be clearly seen from the new carboxilic peaks at 170-176 ppm in the ^{13}C NMR, compared to the original peak at 177 ppm for polylactic acid **31**.

References

S1. M. Chen, V. E. Jackson, A. R. Felmy and D. A. Dixon. *J. Phys. Chem. A,* 2015, **119**, 3419-3428.

S2. M. J. Frisch, G. W. Trucks, H. B. Schlegel, G. E. Scuseria, M. A. Robb, J. R. Cheeseman, G. Scalmani, V. Barone, G. A. Petersson, H. Nakatsuji, X. Li, M. Caricato, A. Marenich, J. Bloino, B. G. Janesko, R. Gomperts, B. Mennucci, H. P. Hratchian, J. V. Ortiz, A. F. Izmaylov, J. L. Sonnenberg, D. Williams-Young, F. Ding, F. Lipparini, F. Egidi, J. Goings, B. Peng, A. Petrone, T. Henderson, D. Ranasinghe, V. G. Zakrzewski, J. Gao, N. Rega, G. Zheng, W. Liang, M. Hada, M. Ehara, K. Toyota, R. Fukuda, J. Hasegawa, M. Ishida, T. Nakajima,Y.; Honda, O. Kitao, H. Nakai, T. Vreven, K. Throssell, J. A. Montgomery Jr., J. E. Peralta, F. Ogliaro, M. Bearpark, J. J. Heyd, E. Brothers, K. N. Kudin, V. N. Staroverov, T. Keith, R. Kobayashi, J. Normand, K. Raghavachari, A. Rendell, J. C. Burant, S. S. Iyengar, J. Tomasi, M. Cossi, J. M. Millam, M. Klene, C. Adamo, R. Cammi, J. W. Ochterski, R. L. Martin, K. Morokuma, O. Farkas, J. B. Foresman, D. J. Fox, Gaussian 09, Revision C.01; Gaussian 16, Revision A.03, Gaussian, Inc., Wallingford CT, 2016.

S3. a) Y. Yang, K. Yao, M. P. Repasky, K. Leswing, R. Abel, B. K. Shoichet and S. V. Jerome. *J. Chem. Theory Comput.,* 2021, **17**, 7106-7119; b) R. A. Friesner, R. B. Murphy, M. P. Repasky, L. L. Frye, J. R. Greenwood, T. A. Halgren, P. C. Sanschagrin and D. T. Mainz. J. Med. Chem., 2006, **49**, 6177–6196; c) T. A. Halgren, R. B. Murphy, R. A. Friesner, H. S. Beard, L. L. Frye, W. T. Pollard and J. L. Banks. *J. Med. Chem.,* 2004, **47**, 1750-1759; d) R. A. Friesner, J. L. Banks, R. B. Murphy, T. A. Halgren, J. J. Klicic, D. T. Mainz, M. P. Repasky, E. H. Knoll, D. E. Shaw, M. Shelley, J. K. Perry, P. Francis and P. S. Shenkin. *J. Med. Chem.,* 2004, **47**, 1739-1749; e) Schrödinger Release 2024-4: Glide, Schrödinger, LLC, New York, NY, 2024.

S4. S. Boys and F. Bernardi. *Mol. Phys.* 1970, **19**, 553-566.
